# Population Structure and Genetic Diversity Analyses Provide New Insight into the Endemic Species *Aster spathulifolius* Maxim. and Its Evolutionary History

**DOI:** 10.3390/plants13010088

**Published:** 2023-12-27

**Authors:** Gurusamy Raman, Kyoung Su Choi, SeonJoo Park

**Affiliations:** 1 Department of Life Sciences, Yeungnam University, Gyeongsan 38541, Gyeongsanbuk-do, Republic of Korea; bioramg@yu.ac.kr; 2 Plant Research Team, Animal and Plant Research Department, Nakdonggang National Institute of Biological Resources, Sangju 37242, Republic of Korea; choiks010@gmail.com

**Keywords:** *Aster spathulifolius*, genetic diversity, population structure, coastal plant evolution, biogeography, phylogenetic analysis

## Abstract

*Aster spathulifolius*, an ecologically significant plant species native to the coastal regions of Korea and Japan, remains understudied in terms of its genetic structure and evolutionary history. In this study, we employed four chloroplast markers and the nuclear ITS region from 15 populations of *A*. *spathulifolius* from both Korea and Japan, including their islands, to unravel the spatial genetic structure, differentiation, gene flow, phylogenetic, and biogeographical relationships. Analysis based on multiple methods identified a low level of genetic diversity, genetic differentiation and gene flow among *A*. *spathulifolius* populations. Network analysis and principal coordinates analysis showed that 15 populations could be divided into two groups: mainland and island. Furthermore, UPGMA, neighbor-net, maximum-likelihood and Bayesian inference-based phylogenetic tree confirmed that these populations formed two distinct clades. Therefore, the island populations might be treated as *A*. *spathulifolius* populations rather than *A*. *oharai* populations. Divergence time analysis estimated the divergence of *A. spathulifolius* lineages approximately 23.09 million years ago, while ancestral area reconstruction analysis suggested Korea as the potential origin, conflicting with alternative scenarios. These findings contribute to a comprehensive understanding of the evolutionary history, genetic structure, and adaptive strategies of *A. spathulifolius* in coastal environments. Our study challenges previous assumptions and underscores the necessity for further population studies to elucidate the intricate dynamics of this distinctive plant species.

## 1. Introduction

The intricate interplay between ecological and genetic processes gives rise to the population genetic structure, a critical aspect in the study of genetic characteristics and dynamics, especially in the context of rare and endangered species with limited geographical ranges [1]. Understanding the genetic structure and gene flow is imperative due to the heightened vulnerability of such populations to damage, and gene flow, recognized as one of the paramount determinants, plays a pivotal role in shaping the genetic makeup of plant populations [2]. Rare and endangered species, constrained by their restricted geographic distribution, face heightened susceptibility to various threats, with habitat fragmentation emerging as a significant concern [1]. Various factors, including plant characteristics, historical events, ecological dynamics, natural selection, human activities, and other influential elements, collectively contribute to the formation of genetic structure within populations [3].

Coastal regions play a pivotal role in fostering biodiversity and ecological dynamics [4], serving as highly productive areas and offering habitats for a diverse array of organisms. The distribution of these organisms can be shaped by a combination of natural and anthropogenic factors [5]. Moreover, coastal plants have been impacted by the variations of repeated glacial periods during the Pleistocene, influenced by climate and sea level changes [6]. Sea level fluctuations, in particular, have exerted a critical influence on genetic diversity and the historical distribution of organisms, coinciding with the regression and transgression of oceans [7,8,9].

In the context of inland East Asia, climate variations during glacial periods have induced alterations in plant populations, revealing putative refugia and migration corridors [10]. The formation of land bridges in response to climate shifts has played a crucial role in shaping the contemporary genetic structure of these populations [11,12,13]. In contrast with inland plants, coastal plants experience the combined effects of glaciation-related changes and oceanic dispersal modes. This mode theoretically influences the genetic patterns of coastal plants across extensive distances and is recognized as a key factor in understanding their genetic diversity [5,7,8,9,14]. Despite the extensive research on the migration processes and historical refugia of inland plants in East Asia [10] and the intriguing biological implications they present, only a limited number of studies have delved into the phylogeography of coastal plants in this region [9].

*Aster spathulifolius*, a member of the Asteraceae family comprising 1535 genera and 23,000 species, holds significance as the second-largest tribe, Astereae [15]. Exclusive to the coastal regions of the southernmost Korean peninsula and southwestern Japan, this species thrives predominantly in rocky seashore habitats [16,17]. Characterized by its alternate spatulate or obovate leaves, inflorescence heads and cypselae fruits, *A*. *spathulifolius* blooms in pale purple or white flowers from July to November and disperses light orange-brown pappus seeds in November, aided by the wind [18]. Recent studies, such as that of Choi et al. [15], which examined the complete chloroplast genome sequence and that of Sivagami and Park [19], who conducted leaf transcriptome analysis, shed light on studies of *A. spathulifolius*. A comprehensive understanding of the origin and evolution of *A. spathulifolius* in the specific context of coastal regions of Korea, Japan and their surrounding islands is lacking. To address this gap, our study focuses on unraveling the genetic diversity of *A. spathulifolius* populations present in the coastal regions of Korea and Japan. Using molecular markers from both chloroplast and nuclear genomes, we aim to assess population structures, haplotype diversity, nucleotide diversity, genetic differentiation, and gene flow in mainland and island habitats. Additionally, we aim to determine whether the island population belongs to *A*. *spathulifolius* or *A*. *oharai*. Phylogenetic reconstructions will elucidate the relationship between Korean and Japanese populations. Divergence time estimation and biogeographical analyses, including ancestral area reconstructions, will provide insights into the evolutionary history and adaptation strategies of *A*. *spathulifolius* in coastal environments. This research contributes valuable knowledge for conservation and management practices, paving the way for a deeper exploration of the ecological and genetic factors shaping coastal plant populations.

## 2. Results

### 2.1. Analysis of Genetic Diversity

A total of 75 samples (*Aster spathulifolius*) from 12 locations in South Korea and 3 locations from Japan were analyzed. *matK*, *ndhF*, *rbcL*, *rpoC1*, internal transcribed spacer 1 (ITS1), 5.8S ribosomal RNA (rRNA) and ITS2 fragments (751, 1381, 1326, 818, 251, 162 and 214 bp) were amplified and uploaded to GenBank.

The concatenated cp genes and nuclear ITS region of all the individuals were compared. A total of 38 variable sites were identified from 75 accessions. For a single population, the average number of haplotypes (h) ranged from 1 (BS, JJI, JJII, ULII, OKI and JPII) to 3 (AN and DDII); haplotype diversity (Hd) within populations ranged from 0 (BS, JJI, JJII, DDI, ULII, OKI and JPII) to 0.7 (AN and DDII); nucleotide diversity (π) ranged from 0 (BS, JJI, JJII, DDI, ULII, OKI and JPII) to 0.00057 (BY); and the average number of nucleotide differences (k) ranged from 0 (BS, JJI, JJII, DDI, ULII, OKI and JPII) to 2.4 (BY), with an average of 0.48 (Table 1). Tajima’s D test yielded a positive value for all populations except (BS, JJI, JJII, DDI, ULII, OKI and JPII). All of the *A. spathulifolius* populations exhibited low haplotype diversity and low nucleotide diversity (Hd = 0.2533; π = 0.000116).

For the mainland population, the highest genetic diversity was found in the AN population (Hd = 0.7; π = 0.00019), while the lowest (Hd = 0; π = 0) was found in the BS and JPII populations. Similarly, in the island population, the highest genetic diversity was observed in the DDII population (Hd = 0.7; π = 0.00029) and the lowest (0) was identified in the JJI, JJII, DDI, ULII and OKI populations. Overall, the genetic diversity of mainland populations is slightly higher than that of island populations (Table 1).

### 2.2. Genetic Differentiation and Genetic Flow

**Figure 1:**
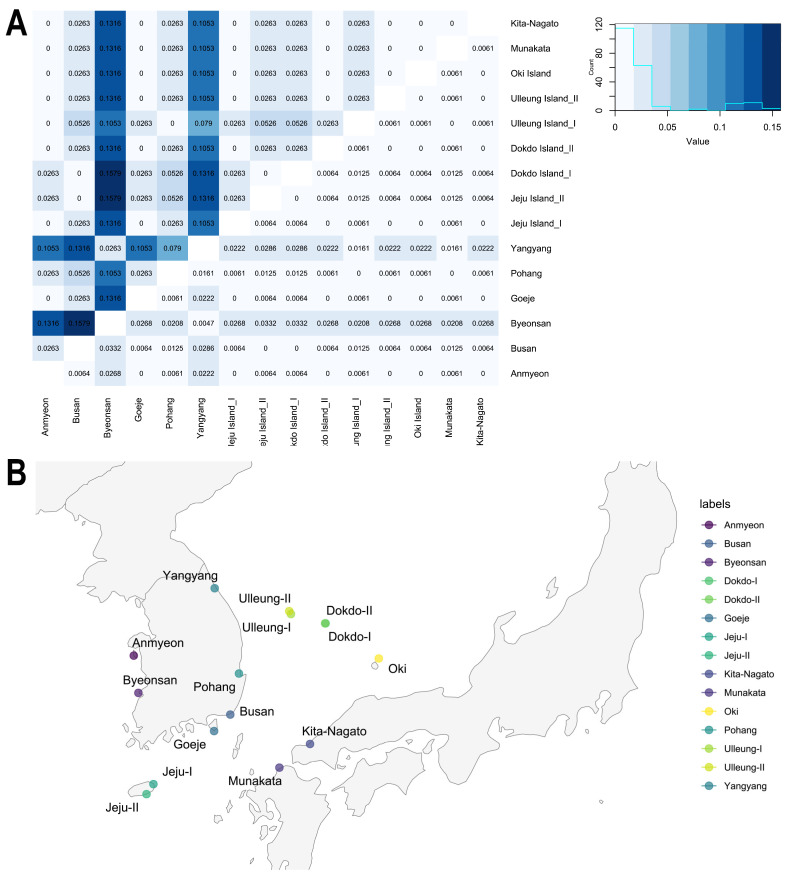
(**A**) Genetic differentiation (*F*_ST_) and gene flow (N*m*) between populations of *A*. *spathulifolius* based on combined genes of chloroplast and nuclear internal transcribed spacer region. *F*_ST_ values are above the diagonal (**right**) and N*m* values are below the diagonal (**left**). (**B**) Geographic map representing the sample collection of *A*. *spathulifolius* populations.

The pairwise *F*_ST_ values, indicative of genetic differentiation, exhibited a range from 0.02632 to 0.1579. Maximum values were discerned particularly between the BS, BY and DDI populations, while the minimum value was consistently identified across all populations (Figure 1). Notably, the BY population displayed heightened genetic differences (0.0332) with other populations, specifically BS, JJII and DDI. Conversely, no genetic differentiation was observed among the AN, BS, GJ, JJI, DDII, ULI, ULII, OKI and JPII populations. However, despite the absence of genetic differentiation, the N*_m_* values indicate a lack of gene flow between these populations (Figure 1A; Supplementary Table S1). The PIC value varied from 0 to 0.4992 (average PIC = 0.29653). The average PIC of the mainland population was 0.2565 and that of the island population was 0.3554.

### 2.3. Network Analysis

**Figure 2:**
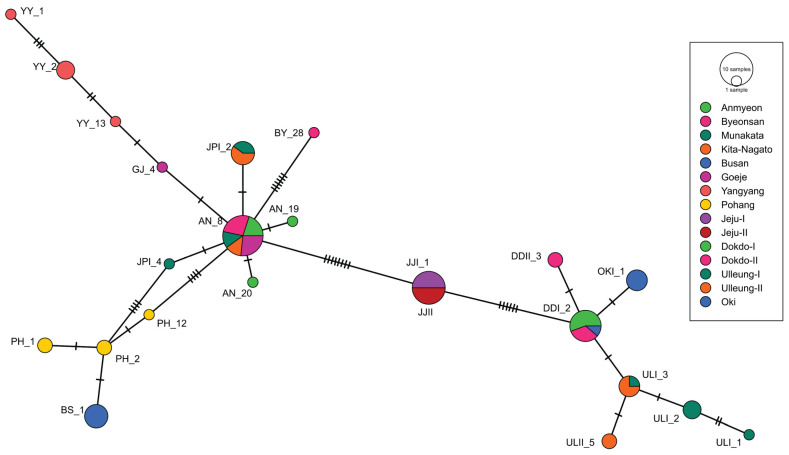
The combined cp genes and nuclear ITS region-based median network map of *A*. *spathulifolius* populations. The size of the circle represents the number of haplotypes. Perpendicular dashes represent mutations. Korean populations: AN—Anmyeon; BS—Busan; BY—Byeonsan; DDI—Dokdo island site I; DDII—Dokdo island site II; GJ—Goeje; JJI—Jeju Island site I; JJII—Jeju Island site II; PH—Pohang; ULI—Ulleung island site I; ULII—Ulleung island site II; YY—Yangyang. Japanese populations: JPI—Munakata; JPII—Kita-Nagato; OKI—Oki Island.

Both TCS and median-joining networks, constructed from the combined cp genes and nuclear ITS region, consistently supported similar phylogenetic relationships. The TCS and median-joining networks based on the combined cp genes and nuclear ITS region highlighted the way in which mainland populations such as YY, PH and BS, remained genetically pure, while other populations exhibited internal mixing (Figure 2). Conversely, in the island populations, Jeju and Ulleung populations showed internal mixing, whereas OKI populations were clustered with Dokdo populations. In contrast, the individual-based analyses of concatenated cp genes (Supplementary Figures S1 and S2) and concatenated ITS region (Supplementary Figures S3 and S4) analyses showed that all of the populations were internally mixed, except for the BS, YY and PH populations, even though these were distinguished into mainland and island clades.

### 2.4. Principal Component Analysis (PCA) and Discriminant Analysis of Principal Components (DAPC)

**Figure 3:**
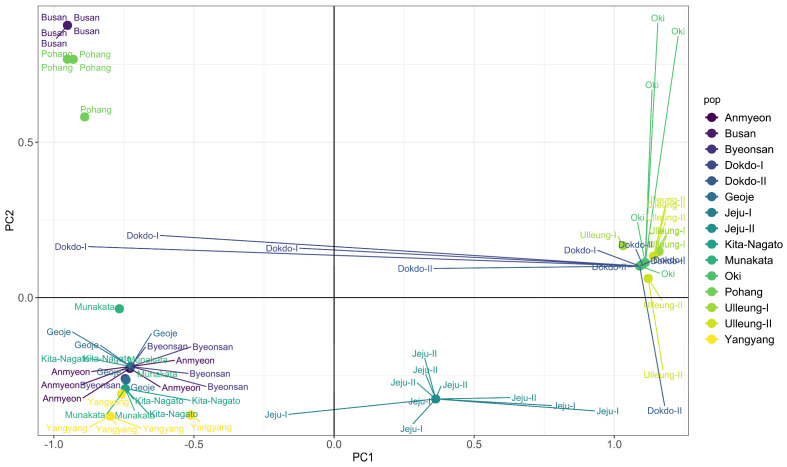
Clustering of combined cp genes and nuclear ITS region of *A*. *spathulifolius* populations based on principal component analysis (PCA). Each point represents an individual color according to their respective population.

We conducted principal component analysis (PCA) to visualize the relationship between the Korean and Japanese populations of *A*. *spathulifolius* using separate datasets for concatenated cp genes (Supplementary Figure S5), concatenated nuclear ITS region (Supplementary Figure S6) and concatenated cp genes and nuclear ITS region (Figure 3). Across all datasets, the *A*. *spathulifolius* populations consistently formed four distinct groups: group A, comprising AN, BY, GJ, YY, JPI and JPII populations; group B, including BS and PH populations; group C, encompassing JJI and JJII; and group D, consisting of DDI, DDII, ULI, ULII and OKI populations.

**Figure 4:**
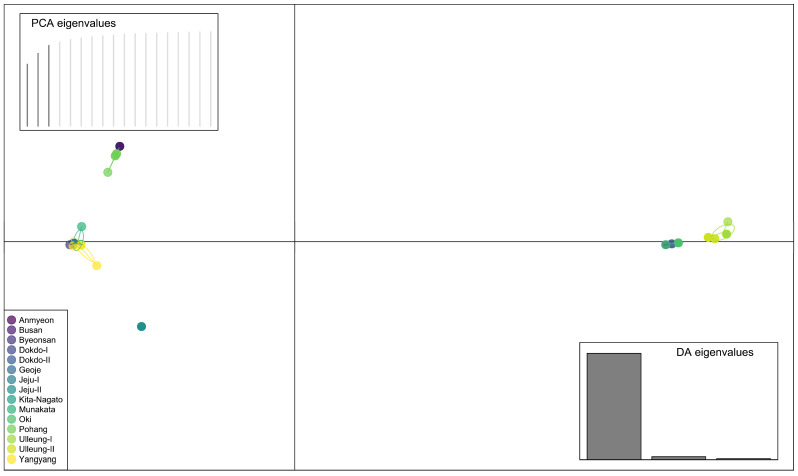
Clustering of combined cp genes and nuclear ITS region of *A*. *spathulifolius* populations based on discriminant principal component analysis (DAPC). Each point represents an individual color according to their respective population.

**Figure 5:**
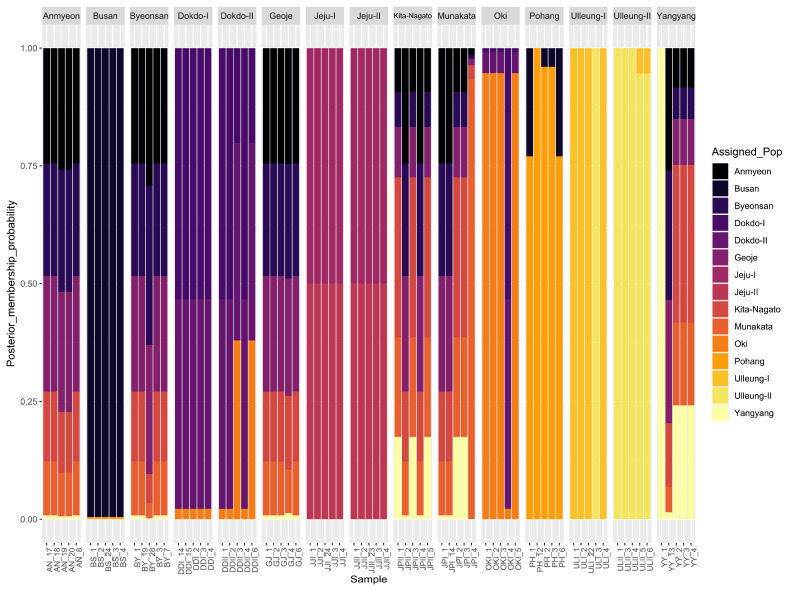
The combined cp genes and nuclear ITS region-based bar plot represents the population membership probability assignments against their original populations of *A*. *spathulifolius*. Each facet represents the original population assignment for each sample.

Additionally, discriminant analysis of the principal component (DAPC) was employed to identify de novo clusters within Korean and Japanese *A*. *spathulifolius* populations (Figure 4; Supplementary Figures S7 and S8). Interestingly, one individual from the JJI population was separated from group C (the Jeju populations). The populations from Jeju, Dokdo and Oki formed one cluster, while the Ulleung populations formed another cluster (Figure 4). DAPC conclusions were congruent with PCA, indicating strong clustering of all individuals of JJI with JJII populations (Jeju populations). Furthermore, we employed compoplot to visually represent the probability of population membership. The color fill in each facet of the compoplot reflects the original population assignments. This analysis brought to light that the Busan (BS), Jeju (JJI and JJII) and Ulleung (ULI and ULII) populations exhibited limited interconnection with other populations. In contrast, populations such as AN, BY, JPI, GI, JPII and YY displayed pronounced interlinkages, specifically with the Dokdo, Jeju and Japan populations (Figure 5; Supplementary Figures S9 and S10).

The population structure of *A*. *spathulifolius* was examined using the model-based method applied in the STRUCTURE software v2.3.4, wherein each individual was assigned a membership coefficient for each cluster. The optimal number of populations (K) was determined following the approach of Evanno et al. [20], and the results exported from STRUCTURE indicate a maximum delta K (ΔK) value of two. This suggests that K = 3 was the most likely value for the *A*. *spathulifolius* populations. With K = 3, *A*. *spathulifolius* population groups exhibited alignment with the taxa of the phylogenetic tree. The STRUCTURE analysis unveiled a partition of *A*. *spathulifolius* populations into two distinct groups: mainland and island groups (Supplementary Figure S11).

### 2.5. Phylogenetic Analysis

**Figure 6:**
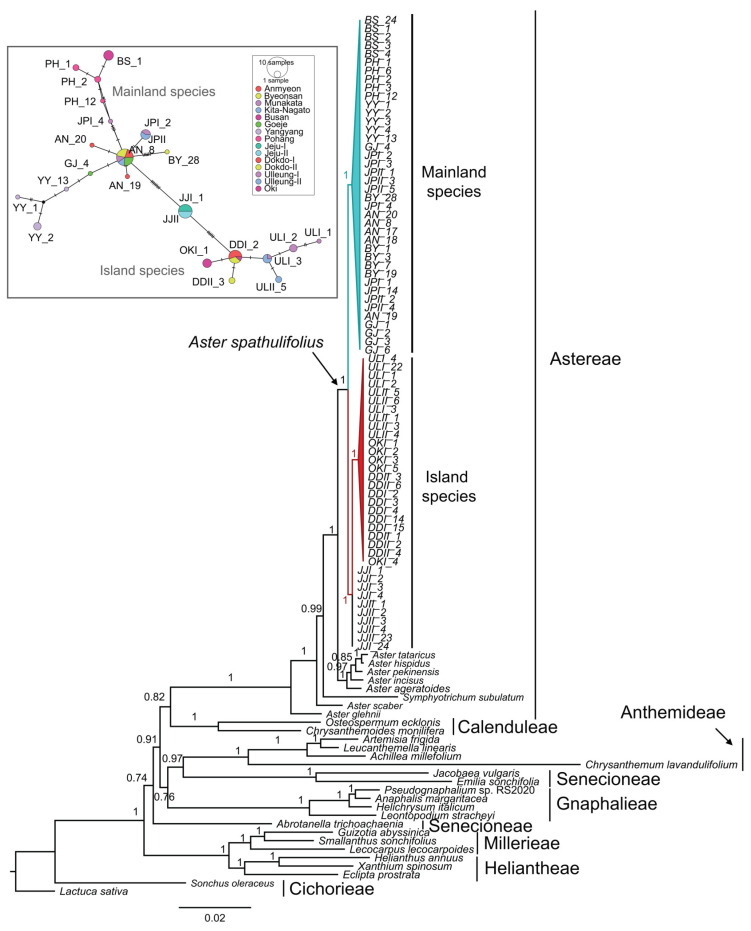
Bayesian Markov chain Monte Carlo (MCMC) inference of the MrBayes phylogenetic tree based on combined cp genes and nuclear ITS region. The gamma model of rate variation and the HKY85 substitution model were utilized for this analysis. The Bayesian tree is depicted with the Bayesian inference posterior probability value given for each node. The inner rectangular box depicts the combined cp genes and nuclear ITS region-based TCS network map of *A*. *spathulifolius* populations. The size of the circle represents the number of haplotypes. Perpendicular dashes represent mutations.

**Figure 7:**
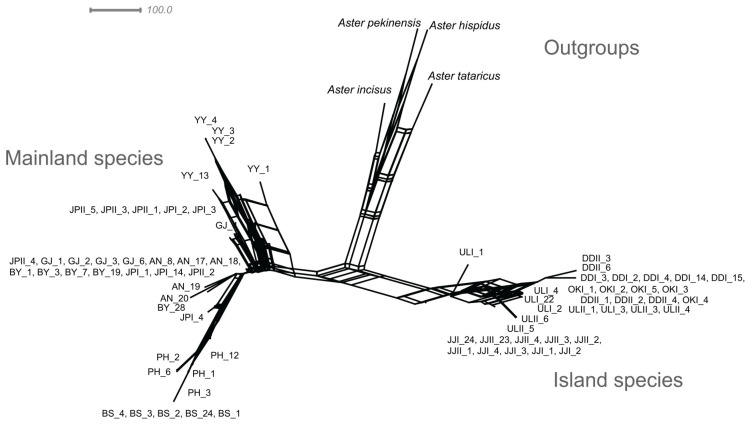
Neighbor-net tree based on combined cp genes and nuclear ITS regions of *A*. *spathulifolius* populations.

Based on the analysis of combined cp genes, nuclear ITS region and the combined dataset comprising both cp genes and nuclear ITS region, the maximum likelihood (ML) (Supplementary Figures S12–S14), Bayesian inference (BI) (Figure 6; Supplementary Figures S15 and S16), UPGMA (Supplementary Figures S17–S19) and neighbor-net (NN) (Figure 7; Supplementary Figures S20 and S21) trees consistently revealed a division into the following primary clades: Clade I (mainland), encompassing populations AN, BS, BY, GJ, PH, YY, JPI and JPII and Clade II (island), comprising populations DDI, DDII, JJI, JJII, OKI, ULI and ULII. Within Clade I, distinct clusters were formed by BS, PH, YY, BY populations, highlighting their individuality. Notably, the GJ and AN populations remained pure, although sharing a single individual with YY and JP populations. The Japanese populations (JPI and JPII) displayed mixing within themselves. Similarly, in Clade II, populations from Jeju (JJI and JJII), Dokdo (DDI and DDII), and Ulleung (ULI and ULII) exhibited internal mixing. One individual from OKI clustered with Dokdo populations. It is noteworthy that ML, BI and NN phylogenetic patterns were consistent across all analyses, and that the identified clades were strongly supported by robust bootstrap values (Figure 6). In contrast, the unrooted UPGMA phylogenetic tree illustrates that, within Clade I (mainland clade), all populations exhibited mixing, except for the BS population (Supplementary Figures S17–S19). Similarly, in Clade II (island), ULI, ULII and DDII populations showed internal mixing. The UPGMA tree also indicates moderated bootstrap values for all clades.

### 2.6. Estimation of Divergence Time

**Figure 8:**
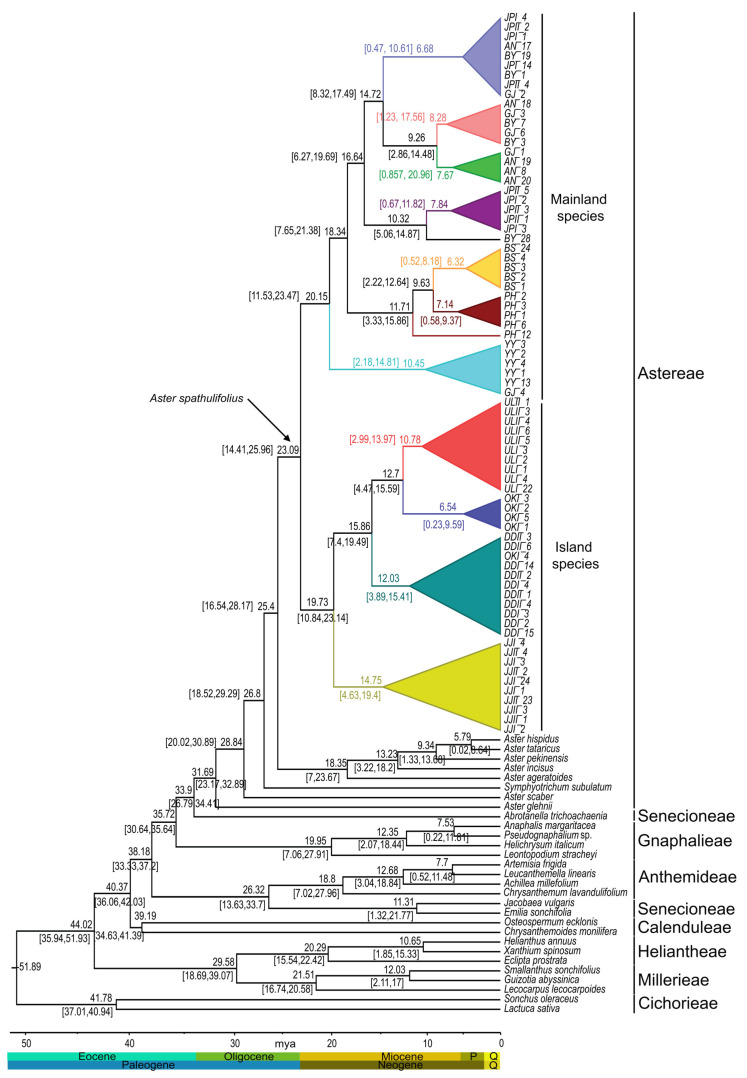
The molecular clock tree depicts the divergent times of Asteraceae. The distribution of age estimates is shown on the branches. Each triangle color in the phylogenetic tree represents a population.

Utilizing concatenated data sets from four cp genes and nuclear ITS region across 104 Asteraceae species, our divergence time analysis indicated that the initial divergence of the Astereae subfamily stem lineage occurred approximately 31.69 million years ago (mya) during the early Oligocene period. This estimation was associated with a 95% higher probability density (HPD) range of 23.17–32.89 mya. Within the subfamily of Astereae, the stem lineage of *A. spathulifolius* species was approximately 23.09 (95% HPD = 14.41–25.96 mya). The *A*. *spathulifolius* cluster was divided into two clades: Clade I consisted of mainland populations of both Korean and Japanese origin and the stem lineage of this clade was dated to 20.15 mya (95% HPD = 11.53–23.47) (Figure 8). Conversely, Clade II included island populations from both Korean and Japanese origins, with the stem lineage estimated at 19.73 mya (95% HPD = 10.84–23.14).

### 2.7. Ancestral Area Reconstruction

**Figure 9:**
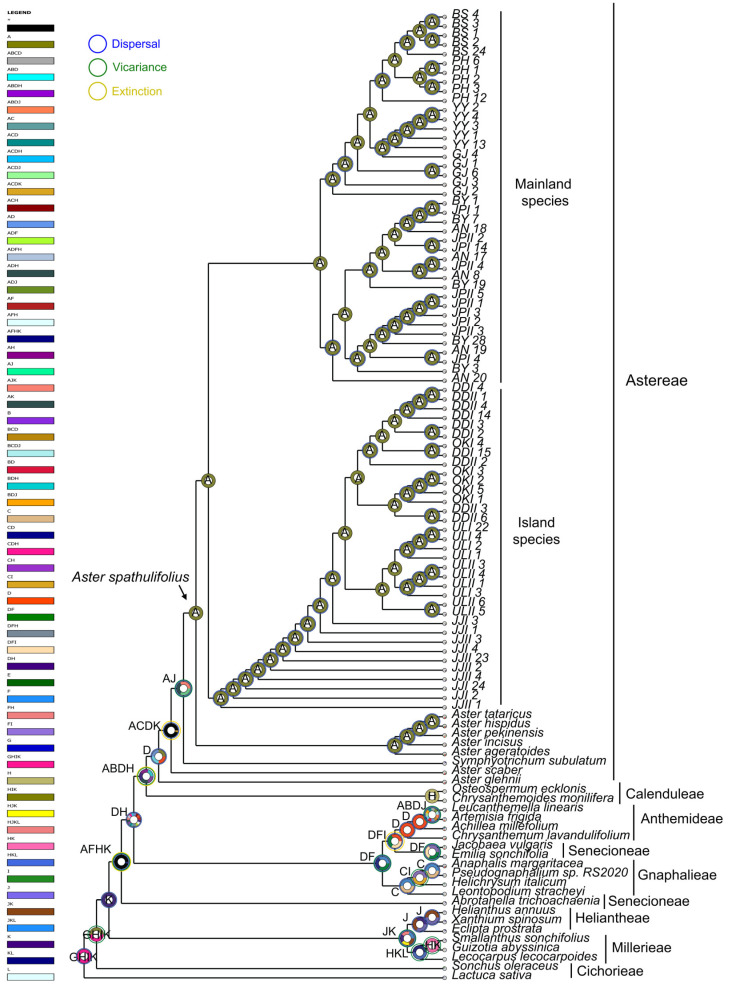
Ancestral geographical ranges inferred with the S-DIVA method. Pie charts indicate the relative probabilities of different ancestral area reconstructions. Geographical regions represented by letters: Korea (A), Japan (B), China (C), Russia and Mongolia (D), central Asia (E), south Asia (F), Middle East Asia (G), Africa (H), Europe (I), North America (J), South America (K), and the Pacific Ocean (L).

The biogeographical history of the *A. spathulifolius* population was systematically examined utilizing a suite of ancestral area reconstruction methodologies, including S-DIVA (Figure 9), DEC (Supplementary Figure S22), S-DEC (Supplementary Figure S23), BBM (Supplementary Figure S24) and BayArea (BA) (Supplementary Figure S25) analyses. The results obtained from S-DIVA analyses propose that the origin of the *A*. *spathulifolius* population lies in Korea, which can be attributed to a dispersal event (Figure 9). However, divergent outcomes were observed in all other analyses, indicating various scenarios wherein both Korea and Japan could potentially be identified as the origin of this species and that involve a combination of vicariance and dispersal events (Supplementary Figures S22–S25).

## 3. Discussion

The significance of spatial genetic structure looms large in the short-term evolutionary dynamics of populations [21]. This structure emerges from the intricate interplay between plant traits and ecological variables, subject to modulation by human activities, historical events, natural selection, and a myriad of other factors [1]. In the context of species featuring a narrow distribution range, the predominant factor influencing small-scale spatial genetic structure is constrained gene flow. Species characterized by limited gene flow tend to exhibit the phenomenon of individual aggregation, thereby manifesting distinct spatial genetic structures within the population [1]. *Aster spathulifolius* belonging to the Astereae subfamily, represents a distinctly narrowly distributed endemic species native to both Korea and Japan. This species is indigenous to the coastal regions of Korea and Japan, including their respective islands [17]. As the predominant species within its distribution area, the abundance and composition of the *A*. *spathulifolius* population significantly influence the overall biodiversity and ecological dynamics in this region. Despite its ecological uniqueness, the genetic structure and the fundamental influencing interrelatedness of *A*. *spathulifolius* have hitherto remained unexplored through genetic data. Although three genomic studies have delved into the genomic structure of *A*. *spathulifolius*, a comprehensive absence of population studies is evident. To our knowledge, this is the first population study of *A*. *spathulifolius* aimed at unraveling the genetic structure, differentiation, gene flow, phylogenetic relationships, and the inherent origin of both Korean and Japanese populations. To achieve this, we employed four plastid genes and the nuclear ITS region, analyzing 12 populations of *A. spathulifolius* from the Korean peninsula and three populations from the coastal regions of Japan.

Through the analyses of both cp genes and nuclear ITS region data, all fifteen populations were categorized into two groups: mainland and island populations. Hd and π are essential indicators of genetic diversity. Various genetic diversity parameters from our study indicated a low level of genetic diversity across all populations (Table 1). The overall populations exhibited high haplotype diversity ranging from 0 to 0.7. However, all of these displayed a low nucleotide diversity (<0.005). In comparison with island populations (Hd = 0.157143; π = 0.000056), mainland populations exhibited higher genetic diversity (Hd = 0.3375; π = 0.000169), surpassing the average genetic diversity per population (Hd = 0.25333; π = 0.000116). Among the fifteen populations, the AN population displayed the highest genetic diversity (Hd = 0.7; π = 0.00019), followed by the BY population (Hd = 0.4; π = 0.00057) (Table 1). Previous studies have suggested that geographical distribution is a factor influencing the genetic diversity of plant species, with species covering a broader range that typically exhibits higher levels of genetic diversity [22]. However, our present study has revealed that both mainland and island populations of Korean and Japanese *A*. *spathulifolius* have notably low genetic diversity levels. Geographic distribution, in this case, does not appear to significantly influence the genetic diversity of *A*. *spathulifolius* populations. Moreover, our current study diverges from previous genetic diversity investigations that employed inter-simple sequence repeat (ISSR) markers [23]. The earlier studies grouped *A*. *spathulifolius* from Ulleung, Dokdo and Oki Islands together, separating them from species on the mainland areas of Korea and Japan [23]. In contrast, our current study utilizing cp genes and the nuclear ITS region, reveals a distinct clade formation, segregating island plants from mainland plants.

The examination of genetic differentiation and gene flow across the fifteen populations revealed no discernible genetic differentiation or gene flow among the various mainland and island populations in Korea and Japan (Figure 1A). This observation was further corroborated by the absence of admixtures in the population STRUCTURE analysis across all populations (Supplementary Figure S11). This finding contrasts with the perspective of Tyagi et al. [24] who considered island populations of *A*. *spathulifolius* as *A*. *oharai*. However, the present study did not identify any genetic differentiation or gene flow in the island population. Furthermore, no morphological differences were observed between the mainland and the island. Consequently, we strongly advocate for the designation of island species as *A*. *spathulifolius* rather than *A*. *oharai*, based on the absence of genetic diversity, genetic differentiation, gene flow and morphological distinctions in our comprehensive studies.

In accordance with ecological theory, it is generally anticipated that small populations of narrowly distributed species will exhibit diminished genetic variation but heightened genetic differentiation among populations [17]. Conversely, our investigation into *A*. *spathulifolius* populations reveals a pattern of low genetic variation and limited gene flow. In consonance with our findings, the earlier research conducted by Maki and Morita [17] on the genetic diversity of *A*. *spathulifolius* within mainland Japan and the islands of both Korea and Japan concluded the absence of discernible genetic diversity distinctions between mainland and island populations of *A*. *spathulifolius*. Therefore, we propose that limited gene flow among mainland populations might have contributed to this lack of genetic differentiation and restricted gene flow among the islands or between the mainland and island populations.

The Korean and Japanese populations of *A*. *spathulifolius* populations were subjected to further analysis through network analyses, UPGMA dendrogram, PCA and DAPC analyses (Figure 2, Figure 3, Figure 4 and Figure 5; Supplementary Figures S2–S10 and S17–S19). All of the analyses were consistent in revealing the formation of a single cluster encompassing both Korean and Japanese mainland populations (AN, BS, BY, GJ, PH, YY, JPI and JPII), while the islands of both Korean and Japanese populations (Jeju, Dokdo, Ulleung, and Oki) constituted another distinct cluster. These results are further supported by neighbor-net and ML and BI phylogenetic analyses, all demonstrating robust cluster formation with strong bootstrap values (Figure 6 and Figure 7; Supplementary Figures S12–S16, S20 and S21).

Tracing the origin of *A*. *spathulifolius* populations utilizing *Lactuca* as an outgroup, molecular clock analysis revealed a monophyletic tree, indicating the initiation of diversification approximately 23.09 mya (95% HPD: 14.41–25.96 mya), signifying the emergence of *A*. *spathulifolius* lineages on the Korean peninsula. This divergence subsequently led to the formation of two clades: one for the mainland areas of the Korean peninsula and Japan, estimated at around 20.15 mya (95% HPD: 11.53–23.47 mya), and another at roughly 19.73 mya (95% HPD: 10.84–23.14 mya) for the islands (Figure 8). Further, biogeographical analyses, employing various ancestral area reconstruction methods—S-DIVA, DEC, S-DEC, BBM and BayArea (BA)—have yielded valuable insights into the evolutionary history and origin of *A*. *spathulifolius*. Consistent findings from S-DIVA analysis suggest that the potential area of this origin of the *A*. *spathulifolius* was in Korea (Figure 9). This implies that the diversification of *A*. *spathulifolius* likely began in Korea, with subsequent events leading to expansion into Japanese regions. However, other analyses (DEC, S-DEC, BBM and BA) present different scenarios regarding the origin, suggesting a possible combined origin in Korea and Japan (Supplementary Figure S22–S25).

Molecular clock analysis suggests that the diversification of the *A*. *spathulifolius* lineage occurred during the early Miocene period, a time when Japan was not separated from Korea. Matsuzaki et al. [25] proposed the origin of Japan around 7–8 mya, and the volcanic islands Dokdo [26], Ulleung [27] and Jeju [28] originated at 2.7–4.6, 1.8 and 0.3–0.6 mya, respectively. We propose that the origin of *A. spathulifolius* is likely situated on the southeast coast of the Korean peninsula, rather than in Japan. This inference is based on an estimated origin timeframe for the Japanese population of around 7.84–6.68 mya (95% HPD: 11.82–0.47 mya), corresponding to the late Miocene to late Quaternary period. Data presented in Figure 8 also suggest a more recent dispersal of *A*. *spathulifolius* to Busan and Pohang. Despite these insights, the exact origin of the species remains elusive.

Speculations regarding the migration of *A*. *spathulifolius* from the continent to Japan or from southernmost Korea to southwestern Japan via Korea/Tsushima land bridges during the Pleistocene might have served as a temporary genetic corridor. Additionally, sea currents are generally considered contributors or barriers to the spread of coastal plants [5]. In our case, the influence of sea currents, including the North Korean current, the East Korean warm current and the Tsushima current, is thought to be involved in facilitating the dispersal of *A. spathulifolius* populations across the coastal regions of southernmost Korea and southwestern Japan. Moreover, alternative propagule dispersal mechanisms, such as wind and migratory birds, are considered potential contributors to the migration of *A*. *spathulifolius* [17]. The pollination strategy of this species by small bees or syrphid flies underscores the limited interpopulation gene flow via pollen, distinguishing it from species pollinated by highly mobile animals like hummingbirds [17]. Despite the potential for substantial genetic variation in a sufficiently large population, the observed low genetic diversity in *A*. *spathulifolius* is attributed to its restricted distribution range, confined to the coastal regions of southernmost Korea and southwestern Japan. The comprehensive understanding of the evolutionary history and dispersal patterns of *A*. *spathulifolius* contributes to an enriched knowledge of how the species has adapted to specific ecological niches in coastal environments. This knowledge provides insights into the factors that have influenced the genetic structure, distribution and adaptive strategies of *A*. *spathulifolius* populations in these coastal habitats.

## 4. Materials and Methods

### 4.1. Sample Collection

In this study, we aimed to investigate the geographic distribution of *Aster spathulifolius* in South Korea and Japan. We collected a total of 450 individuals from all 15 populations (30 individuals from each population) (Figure 1B), with the abbreviation of place names for populations shown in Supplementary Table S2. To avoid clone sampling, we randomly chose five individuals from each population and collected each plant at a minimum distance of two meters from each other. Additionally, we collected seven other outgroup species of *Aster* and one *Sonchus oleraceus* species for this study. All samples were identified by Prof. SeonJoo Park from the Department of Life Sciences at Yeungnam University, South Korea, and a voucher specimen was deposited in the Yeungnam University Herbarium, Gyeongsan, South Korea. We collected healthy leaves from 458 individuals, which were separately stored with a silica gel pack and then taken back to the lab, where they were frozen at −80 °C until DNA extraction.

### 4.2. DNA Extraction, Amplification, Sequencing and Variant Calling

Total genomic DNA was extracted from fresh leaves of all populations using the Qiagen DNeasy Plant Mini Kit (Qiagen Co., Hilden, Germany). The quality and quantity of DNA were determined by agarose gel electrophoresis and UV–vis spectrophotometry. Amplification of the specific chloroplast genes, *matK*, *ndh*, *rbcL*, and *rpoC1* and nuclear internal transcribed spacer (ITS) regions, such as ITS 1, 5.8S ribosomal RNA (rRNA) and ITS 2, was conducted using genomic DNA of each population of plant material as a template with gene-specific primers (Supplementary Table S3). All gene-specific primers were designed using Primer3 [29] in Geneious Prime v2023.2.1 (Biomatters, Auckland, New Zealand) and PCR analysis was carried out as described previously [30]. All the sequencing reads were assembled by de novo using the Geneious assembler under the default settings of Geneious Prime v2023.2.1. All of the cp genes and the nuclear ITS region were aligned individually using MAFFT v7.017 [31]. All sequences have been deposited in GenBank (accession numbers: OR889014–OR889096; OR908446–OR908777). The variant call format (VCF) was generated using the R package SNP-sites v2.5.0 [32]. The geographic map was created using the R package “rworldmap” (https://cran.r-project.org/web/packages/rworldmap/, accessed on 18 November 2023) and “ggplot2” (https://cran.r-project.org/web/packages/ggplot2/, accessed on 18 November 2023).

### 4.3. Genetic Diversity Analysis

Genetic diversity was estimated by the number of polymorphic sites (S), the number of haplotypes (h), haplotype diversity (Hd), nucleotide diversity (π), and the average number of nucleotide differences (κ) were analyzed for concatenated cp and nuclear genes, using DnaSP v6.12.03 [33].

### 4.4. Principle Component Analysis

Principle component analysis (PCA) was constructed using the R package poppr v2.9.3 [34] to determine the genetic relationships of mainland and island populations of *A*. *spathulifolius* accessions. Discriminant analysis of population components (DAPC) was carried out to evaluate the population structure of *A*. *spathulifolius* populations using the package poppr v2.9.3 in R. All graphs and plots were generated using the ggplot2 v3.4.4 and tidyvr packages v1.3.0 in R.

### 4.5. Population Structure Analysis

PopART v1.7 [35] was used to create a statistical median-joining and TCS network to infer haplotype relationships from the concatenated cp, ITS, and both cp and ITS alignment sequences, individually. Population structure was assessed using STRUCTURE v2.3.4 with Bayesian model-based cluster analysis based on admixture and correlated allele frequency models [36]. For each value of K from 2 to 10, a 10,000 iteration burn-in period followed by 100,000 Markov chain Monte Carlo (MCMC) was used in each run. The most appropriate number of genetic clusters was derived using delta K [20].

### 4.6. Genetic Differentiation and Genetic Flow

Genepop program v4.7 in R package was used to analyze pairwise fixation (*F*_ST_) and gene flow indices (Nm) of each SNP locus in all individuals [37]. The mean number of alleles (Na), the effective number of alleles (Ne), observed heterozygosities (Ho), expected heterozygosities (He), and the polymorphism information content (PIC) were calculated according to the formula. The *F*_ST_ values were calculated using the formula *F*_ST_ = (variation between populations − variation within populations)/variation between populations and Nm (gene flow), which were calculated using the formula Nm = (1 − *F*_ST_)/4 *F*_ST_.

### 4.7. Phylogenetic Analyses

In addition to the 83 individuals used in this study, 21 other species of Asteraceae sequences were retrieved from the NCBI. Subsequently, we prepared three data sets as concatenated (i) four cp genes (*matK*, *rbcL*, *ndhF* and *rpoC1*), (ii) nuclear ITS region (ITS 1, 5.8S rRNA and ITS 2), and (iii) concatenated both cp genes and nuclear ITS region of all the individuals, separately. The species, *Lactuca sativa* was used as the outgroup.

To create a splitstree, we initially converted the filtered VCF file into a nexus format using the vcf2phylip.py script. Subsequently, the generated nexus file was employed to construct an unrooted splitstree through the neighbor-net method using SPlitsTree software v4.17.0 [38]. Hierarchical clustering was analyzed using UPGMA by calculating a pairwise dissimilarity matrix between the genotypes. The Nei coefficient was employed to estimate the genetic distance between genotypes, and 1000 bootstraps were applied [39]. UPGMA-based population structure analysis was performed using the poppr package v2.9.3 [34], and genetic distance was estimated using the ape package v5.7.1 [40].

The jModelTest 2 v0.1.10 program was used to find the best nucleotide substitution model for all the concatenated genes using optimized parameters [41]. All three sets of sequences were aligned separately using MAFFT v7.017 [31] through Geneious Prime v2023.2.1. The aligned gene sequences were saved in PHYLIP format using Clustal X v2.0 [42] and used to generate a phylogenetic tree. Maximum likelihood (ML) analysis was performed with RaxML v8.0 [43] using the general time-reversible invariant gamma-sites (GTR + I + G) nucleotide substitution model with the default parameters. Statistics support for the branches was calculated by rapid analysis with 1000 bootstrap replicates. Additionally, for Bayesian analysis, we analyzed the combined genes based on the GTR + I + G model for rate variation using MrBayes v3.2.6 [44].

### 4.8. Divergence Dating Analyses

To analyze the divergence times of the *A*. *spathulifolius* populations of Japan and Korea, a BI approach with Markov chain Monte Carlo (MCMC) sampling was performed using BEAST v2.5 [45], with minor modifications [30]. In the BEAST analysis, a relaxed-clock log-normal model was employed. The MCMC chain had 800 million steps, with sampling conducted every 1000 generations, and a 10% burn-in phase was utilized. A GTR nucleotide substitution model was applied, with a gamma distribution with four rate categories. Using a Yule prior, diversification times and credibility intervals were evaluated. The assessment of the sample size was implemented by applying Tracer v1.6 analysis software [45].

For the divergence time analysis, seven calibration points were utilized. The calibration for Astereae and Gnaphalieae lineages was set at 32.1 million years ago (mya), while Senecioneae and Anthemideae lineages were calibrated at 35.7 mya. Additionally, the Senecioneae, Anthemideae and Astereae lineages were calibrated at 36.2 mya and the Calenduleae, Heliantheae, Heliantheae and Millerieae and Cichorieae lineages were calibrated at 37.6, 17.5, 35.7 and 39.2 mya, respectively [46]. All calibrations were established with 95% confidence intervals and incorporated using a log-normal distribution. Following the MCMC analysis, TreeAnnotator v2.1.2 was employed to generate a maximum clade credibility (MCC) tree [45].

### 4.9. Biogeographical Analyses

Biogeographic data for Asteraceae species were extracted from the Plants of the World Online (POWO) database, maintained by the Royal Botanic Gardens, Kew, UK (https://powo.science.kew.org, accessed on 01 December 2023). The distribution range of Asteraceae species was delineated into twelve regions: Korea (A), Japan (B), China (C), Russia and Mongolia (D), central Asia (E), south Asia (F), Middle East Asia (G), Africa (H), Europe (I), North America (J), South America (K), and the Pacific Ocean (L).

To elucidate ancestral areas and assess the spatial patterns of geographic diversification, a repertoire of analytical methods was applied. These encompassed statistical dispersal-vicariance analysis (S-DIVA), the Bayesian binary method (BBM), dispersal-extinction-cladogenesis (DEC), the recently modified statistic DEC (S-DEC), and BayArea analysis. All analyses were executed using the Reconstruct Ancestral State in Phylogenies (RASP) v4.2 software [47]. Additionally, the BioGeoBEARS package was employed to select the most fitting model for our investigation.

In these analyses, a concatenated dataset comprising cp genes and nuclear spacer region underwent scrutiny using BEAST software v2.5, yielding a set of 100,000 trees from the MCMC output.

## 5. Conclusions

In conclusion, this comprehensive study illuminates the genetic characteristics, population structure, and evolutionary history of *Aster spathulifolius*, a species endemic to the coastal regions of Korea and Japan. The findings reveal low genetic diversity across populations, distinct clusters corresponding to mainland and island habitats, and limited genetic differentiation and gene flow among populations. Therefore, the island populations might be treated as *A*. *spathulifolius* rather than *A*. *oharai*. Phylogenetic analyses revealed that populations of *A*. *spathulifolius* formed two distinct clades, namely mainland and island clades, and molecular clock analysis suggested the divergent lineage of *A*. *spathulifolius* during the early Miocene. Biogeographical analyses suggest Korea as the likely origin, with subsequent expansion into Japanese regions. The study contributes to a deeper understanding of the *A*. *spathulifolius* adaptation to specific ecological niches and holds implications for conservation strategies and sustainable management practices. Overall, this research lays the groundwork for further exploration of the ecological and genetic factors influencing coastal plant populations.

## Figures and Tables

**Table 1:** Nucleotide diversity, haplotype diversity and minimum number of recombination events of the *A*. *spathulifolius* populations.

	Populations																	
	Mainland Populations								Island Populations							Average/ Population	Mainland Populations	Island Populations
	AN	BS	BY	GC	PH	YY	JPI	JPII	JJI	JJII	DDI	DDII	ULI	ULII	OKI			
S	2	0	6	1	1	3	1	0	0	0	0	3	1	0	0	1.2	1.75	0.571429
h	3	1	2	2	2	2	2	1	1	1	1	3	2	1	1	1.666	1.875	1.428571
Hd	0.700	0	0.4	0.4	0.4	0.4	0.4	0	0	0	0	0.7	0.4	0	0	0.25333	0.3375	0.157143
π	0.00019	0	0.00057	0.0001	0.0001	0.00029	0.0001	0	0	0	0	0.00029	0.0001	0	0	0.000116	0.000169	0.000056
κ	0.8	0	2.4	0.4	0.4	1.2	0.4	0	0	0	0	1.2	0.4	0	0	0.48	0.7	0.228571
Tajima’s D	0.96	0	2.880	0.48		1.44	0.48	0	0	0	0	1.44	0.48	0	0	0.582857	0.891429	0.274286

S—number of polymorphic sites; h—number of haplotypes; Hd—haplotype diversity; π—nucleotide diversity; κ—average number of nucleotide differences.

## Data Availability

Data is contained within the article and supplementary materials.

## Supplementary Materials

The following supporting information can be downloaded at: https://www.mdpi.com/article/10.3390/plants13010088/s1, Table S1: The efficient allelic number (Ne), observed heterozygosity (Ho), expected heterozygosity (He), polymorphism information content (PIC) and gene flow of *A*. *spathulifolius* populations. Table S2: Isolation sites and geographical locations of *Aster spathulifolius*. Table S3: Primers and amplification conditions used in the study. Figure S1: The combined cp genes-based median network map of *A*. *spathulifolius* populations. The size of the circle represents the number of haplotypes. Perpendicular dashes represent mutations. Figure S2: The combined cp genes-based TCS network map of *A*. *spathulifolius* populations. The size of the circle represents the number of haplotypes. Perpendicular dashes represent mutations. Figure S3: The combined nuclear ITS region-based median network map of *A*. *spathulifolius* populations. The size of the circle represents the number of haplotypes. Perpendicular dashes represent mutations. Figure S4: The combined nuclear ITS region-based TCS network map of *A*. *spathulifolius* populations. The size of the circle represents the number of haplotypes. Perpendicular dashes represent mutations. Figure S5: Clustering of combined cp genes of *A*. *spathulifolius* populations based on principal component analysis (PCA). Each point represents an individual color according to their respective population. Figure S6: Clustering of combined ITS region of *A*. *spathulifolius* populations based on principal component analysis (PCA). Each point represents an individual color according to their respective population. Figure S7: Clustering of combined cp genes of *A*. *spathulifolius* populations based on discriminant principal component analysis (DAPC). Each point represents an individual color according to their respective population. Figure S8: Clustering of combined nuclear ITS region of *A*. *spathulifolius* populations based on discriminant principal component analysis (DAPC). Each point represents an individual color according to their respective population. Figure S9: The combined cp genes-based bar plot represents the population membership probability assignments against their original populations of *A*. *spathulifolius*. Each facet represents the original population assignment for each sample. Figure S10: The combined nuclear ITS region-based bar plot represents the population membership probability assignments against their original populations of *A*. *spathulifolius*. Each facet represents the original population assignment for each sample. Figure S11: The STRUCTURE analysis of *A*. *spathulifolius* populations based on K = 3 value. Figure S12: Maximum likelihood (ML) phylogenetic tree based on combined cp genes and nuclear ITS region, with 1000 bootstrap replicates to assess branch support. Figure S13: Maximum likelihood (ML) phylogenetic tree based on combined cp genes, with 1000 bootstrap replicates to assess branch support. Figure S14: Maximum likelihood (ML) phylogenetic tree based on combined nuclear ITS region, with 1000 bootstrap replicates to assess branch support. Figure S15: Bayesian Markov chain Monte Carlo (MCMC) inference of the MrBayes phylogenetic tree based on combined cp genes. The gamma model of rate variation and the HKY85 substitution model were utilized for this analysis. The Bayesian tree is depicted with the Bayesian inference posterior probability value given for each node. Figure S16: Bayesian Markov chain Monte Carlo (MCMC) inference of the MrBayes phylogenetic tree based on combined nuclear ITS region. The gamma model of rate variation and the HKY85 substitution model were utilized for this analysis. The Bayesian tree is depicted with the Bayesian inference posterior probability value given for each node. Figure S17: Distance-based UPGMA phylogenetic tree using combined cp genes and nuclear ITS region., with 1000 bootstrap replicates to assess branch support. Figure S18: Distance-based UPGMA phylogenetic tree using combined cp genes, with 1000 bootstrap replicates to assess branch support. Figure S19: Distance-based UPGMA phylogenetic tree using combined nuclear ITS region, with 1000 bootstrap replicates to assess branch support. Figure S20: Neighbor-net tree based on combined cp genes of *A*. *spathulifolius* populations. Figure S21: Neighbor-net tree based on combined nuclear ITS regions of *A*. *spathulifolius* populations. Figure S22: The DEC method for inferring ancestral geographical ranges within the Asteraceae family relies on utilizing simplified BEAST and MrBayes phylogenetic trees. At various nodes in the phylogeny, pie charts are employed to visually convey the relative likelihoods of different ancestral area reconstructions. Geographical regions represented by letters: Korea (A), Japan (B), China (C), Russia and Mongolia (D), central Asia (E), south Asia (F), Middle East Asia (G), Africa (H), Europe (I), North America (J), South America (K), and the Pacific Ocean (L). Figure S23: The S-DEC method for inferring ancestral geographical ranges within the Asteraceae family relies on utilizing simplified BEAST and MrBayes phylogenetic trees. At various nodes in the phylogeny, pie charts are employed to visually convey the relative likelihoods of different ancestral area reconstructions. Geographical regions represented by letters: Korea (A), Japan (B), China (C), Russia and Mongolia (D), central Asia (E), south Asia (F), Middle East Asia (G), Africa (H), Europe (I), North America (J), South America (K), and the Pacific Ocean (L). Figure S24: The BBM method for inferring ancestral geographical ranges within the Asteraceae family relies on utilizing simplified BEAST and MrBayes phylogenetic trees. At various nodes in the phylogeny, pie charts are employed to visually convey the relative likelihoods of different ancestral area reconstructions. Geographical regions represented by letters: Korea (A), Japan (B), China (C), Russia and Mongolia (D), central Asia (E), south Asia (F), Middle East Asia (G), Africa (H), Europe (I), North America (J), South America (K), and the Pacific Ocean (L). Figure S25: The BayArea method for inferring ancestral geographical ranges within the Asteraceae family relies on utilizing simplified BEAST and MrBayes phylogenetic trees. At various nodes in the phylogeny, pie charts are employed to visually convey the relative likelihoods of different ancestral area reconstructions. Geographical regions represented by letters: Korea (A), Japan (B), China (C), Russia and Mongolia (D), central Asia (E), south Asia (F), Middle East Asia (G), Africa (H), Europe (I), North America (J), South America (K), and the Pacific Ocean (L).
