# Supplementary material for: Population Structure and Genetic Diversity Analyses Provide New Insight into the Endemic Species Aster spathulifolius Maxim. and Its Evolutionary History
Source: Plants (Basel). 2023 Dec 27;13(1):88. doi: 10.3390/plants13010088 (PMC10780962; doi:10.3390/plants13010088)
Supplement: Supplementary file 1 [file plants-13-00088-s001.zip › plants-2584093-supplementary/Supplementary_Files/Supplementary Table S1.pdf]

Supplementary Table S1: The efficient allelic number (Ne), observed heterozygosity (Ho), expected heterozygosity (He), polymorphism information content (PIC) and gene flow of *A. spathulifolius* populations.

Sl. No.	Population	Na	Ne	Ho	He	PIC	F_{ST}	Nm
1.	AN	2	0.5	1	0.32	0.4352	0.97368	0.006407
2.	BS	0	0	0	0	0	1	0
3.	BY	2	0.5	1	0.32	0.4352	0.84210	0.033242
4.	GJ	2	0.5	1	0.32	0.4352	0.97368	0.006407
5.	PH	2	0.5	1	0.4	0.4672	0.94738	0.012463
6.	YY	2	0.5	1	0.32	0.4352	0.86842	0.028567
7.	JJI	0	0	0	0	0	0.97368	0.006407
8.	JJII	0	0	0	0	0	1	0
9.	DDI	0	0	0	0	0	1	0
10.	DDII	2	0.5	1	0.48	0.4992	0.97368	0.006407
11.	ULI	2	0.5	1	0.32	0.4352	0.94738	0.012463
12.	ULII	2	0.5	1	0.32	0.4352	0.97368	0.006407
13.	OKI	2	0.5	1	0.32	0.4352	0.97368	0.006407
14.	JPI	2	0.5	1	0.32	0.4352	0.94738	0.012463
15.	JPII	0	0	0	0	0	0.97368	0.006407
Average		1.33	0.33	0.666	0.22933	0.29653	0.95789	0.0096
Mainland		2	0.5	1	0.181125	0.2565403	0.5	0.0625
Island		2	0.5	1	0.276044	0.3555438	0.63157	0.058172

Mean number of alleles (Na); Effective number of alleles (Ne); Observed heterozygosity (Ho); Expected heterozygosity (He); Polymorphism Information Content (PIC); F_{ST} = (Variation between populations – variation within populations)/variation between populations; Nm gene flow = $(1-F_{ST})/4F_{ST}$
