# Supplementary material for: Population Structure and Genetic Diversity Analyses Provide New Insight into the Endemic Species Aster spathulifolius Maxim. and Its Evolutionary History
Source: Plants (Basel). 2023 Dec 27;13(1):88. doi: 10.3390/plants13010088 (PMC10780962; doi:10.3390/plants13010088)
Supplement: Supplementary file 1 [file plants-13-00088-s001.zip › plants-2584093-supplementary/Supplementary_Files/Supplementary Table S2.pdf]

Supplementary Table S2: Isolation sites and geographical locations of *Aster spathulifolius*.

Sl. No.	Population	Geographical Location	Abbreviation	Sample size
**Korea**				
1.	Anmyeon Island, Chungcheongnam-do, South Korea	36°29'N, 126°20'E	AN	5
2.	Busan, South Korea	35°07'N, 129°07'E	BS	5
3.	Byeonsan, Jeollabuk-do, Korea	35°37'N, 126°28'E	BY	5
4.	Dokdo Island site I, Dongdo, Gyeongsangbuk-do, South Korea	37°14'N, 131°52'E	DDI	5
5.	Dokdo Island site II, Dongdo, Gyeongsangbuk-do, South Korea	37°14'N, 131°51'E	DDII	5
6.	Goeje Island, Gyeongsangnam-do, South Korea	34°44'N, 128°39'E	GJ	5
7.	Jeju Island site I, Jeju-do, South Korea	33°30'N, 126°54'E	JJI	5
8.	Jeju Island site II, Seogwipo, JeJu-do, South Korea	33°16'N, 126°42'E	JJII	5
9.	Pohang, Gyeongsanbuk-do, South Korea	36°04'N, 129°22'E	PH	5
10.	Ulleung Island site I, Sadong-ri, Gyeongsangbuk-do, South Korea	37°27'N, 130°52'E	ULI	5
11.	Ulleung Island site II, Hyeongpo-ri, Gyeongsangbuk-do, South Korea	37°31'N, 130°49'E	ULII	5
12.	Yangyang, Gangwon-do, South Korea	38°03'N, 128°40'E	YY	5
**Japan**				
13.	Munakata, Fukuoka, Japan	33°53'N, 130°32'E	JPI	5
14.	Kita-Nagato coast quasi National Park, Yamaguchi, Japan	34°26'N, 131°25'E	JPII	5
15.	Oki Island, Shimane, Japan	36°25'N, 133°24'E	OKI	5
