# Supplementary material for: Population Structure and Genetic Diversity Analyses Provide New Insight into the Endemic Species Aster spathulifolius Maxim. and Its Evolutionary History
Source: Plants (Basel). 2023 Dec 27;13(1):88. doi: 10.3390/plants13010088 (PMC10780962; doi:10.3390/plants13010088)
Supplement: Supplementary file 1 [file plants-13-00088-s001.zip › plants-2584093-supplementary/Supplementary_Files/Supplementary Table S3.pdf]

Supplementary Table S3 Primers and amplification conditions used in the study.

Gene-specific primer name	Gene-specific primer sequence	Amplification conditions
rbcL		
71F	5'-TGCCAAGCCTCGCT CCAA-3'	Initial denaturation 94°C for 5 min, 30 cycles of 94°C for 40 s, 52°C for 40 s, and 72°C for 60 s. Final extension at 72°C for 5 min.
1379R	5'-CGCGTTGTTCCTCGTTCAT-3'	
matK		
427F	5'-CGCACGGATCCTTTCAGAATGTC-3'	Initial denaturation 94°C for 5 min, 30 cycles of 94°C for 40 s, 54°C for 40 s, and 72°C for 60 s. Final extension at 72°C for 5 min.
1248R	5'-GCCACGCCAAGCTTCACGCTCA-3'	
rpoC1		
*rpoC1*F	5'-GAGGACGTCGAAGACCACCA-3'	Initial denaturation 94°C for 5 min, 30 cycles of 94°C for 30 s, 52°C for 40 s, and 72°C for 60 s. Final extension at 72°C for 5 min.
*rpoC1*R	5'-ACC GCAGCATCGTGTATGAG-3'	
ndhF		
*ndh*1F	5'-CGGAGCATGGACCCCCAGC-3'	Initial denaturation 94°C for 5 min, 30 cycles of 94°C for 30 s, 48°C for 40 s, and 72°C for 60 s. Final extension at 72°C for 5 min.
*ndh*1R	5'-GTGCCCGATATTGGTCACCGAG-3'	
*ndh*2F	5'-GTGTTCTTCGACTTCCTCG-3'	Initial denaturation 94°C for 5 min, 30 cycles of 94°C for 40 s, 51°C for 40 s, and 72°C for 60 s. Final extension at 72°C for 5 min.
*ndh*2R	5'-TGCCGGTTCGCGAGCCAG-3'	
*ndh*3F	5'-GTGTTCTTCGACTTCCTCG-3'	Initial denaturation 94°C for 5 min, 30 cycles of 94°C for 40 s, 52°C for 40 s, and 72°C for 60 s. Final extension at 72°C for 5 min.
*ndh*3R	5'-TGCCGGTTCGCGAGCCAG-3'	
**Internal Transcribed spacer (ITS1, 5.8S rRNA and ITS2)**		
ITS4	5'-TCCTCCGCCTTATTGATATGC-3'	Initial denaturation 94°C for 5 min, 30 cycles of 94°C for 40 s, 56°C for 40 s, and 72°C for 60 s. Final extension at 72°C for 5 min.
ITS5	5'-GGAAGTAAAAGTCGTAACAAGG-3'	
