# Supplementary figures and images for: Population Structure and Genetic Diversity Analyses Provide New Insight into the Endemic Species Aster spathulifolius Maxim. and Its Evolutionary History
Source: Plants (Basel). 2023 Dec 27;13(1):88. doi: 10.3390/plants13010088 (PMC10780962; doi:10.3390/plants13010088)

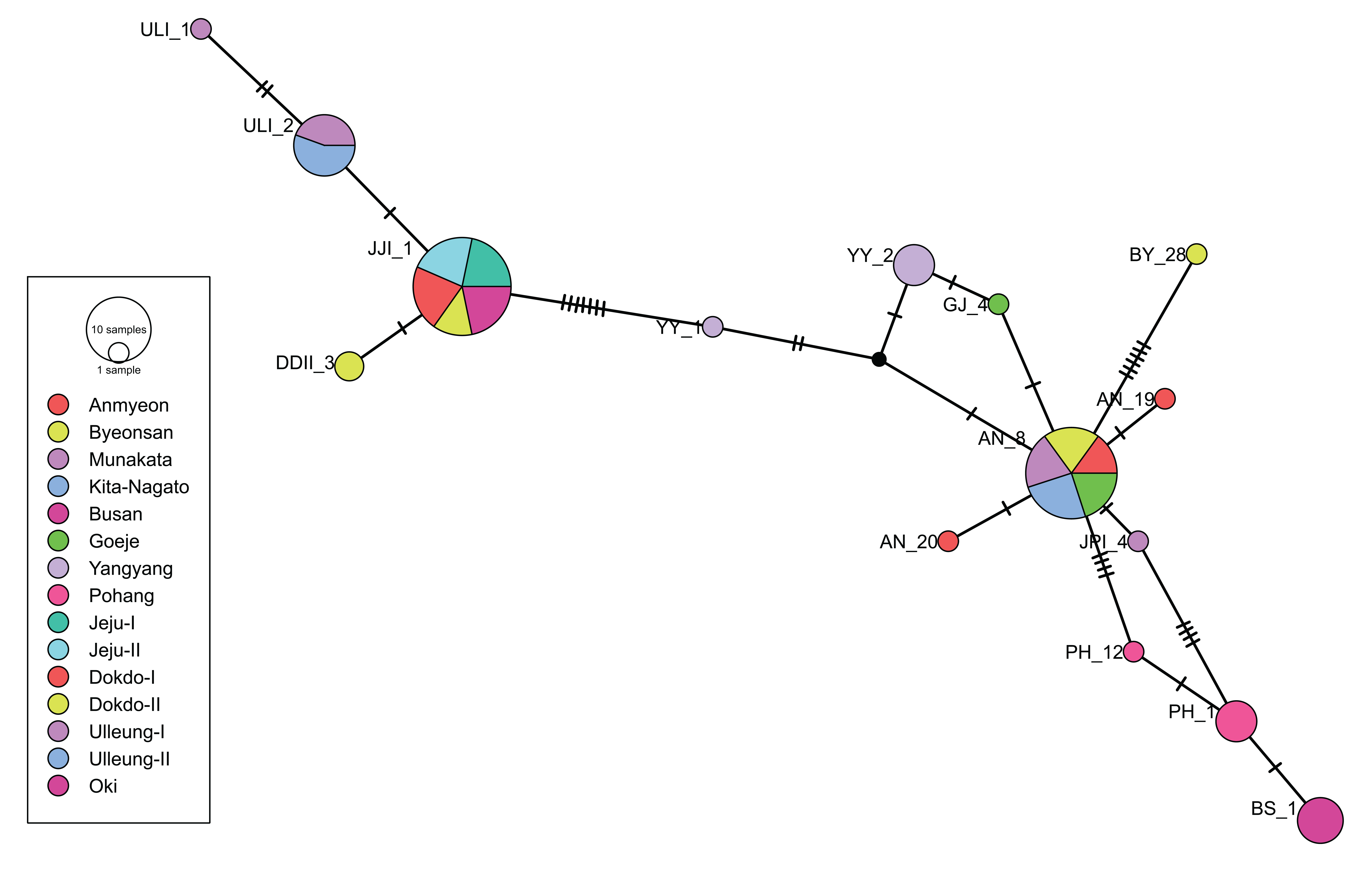

Supplement: Supplementary file 1 [file plants-13-00088-s001.zip › plants-2584093-supplementary/Supplementary_Files/Supplementary Figure S1.tif]

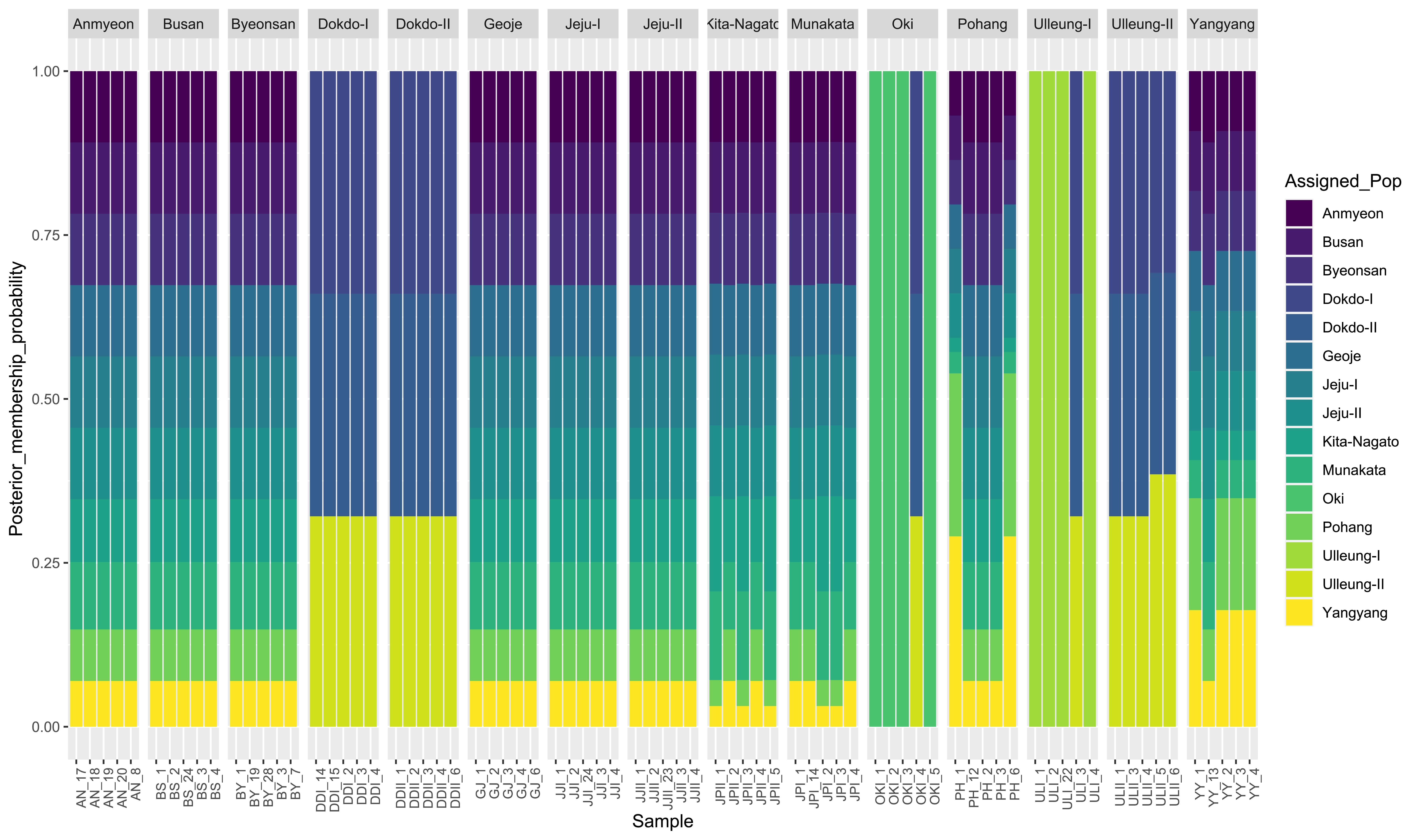

Supplement: Supplementary file 1 [file plants-13-00088-s001.zip › plants-2584093-supplementary/Supplementary_Files/Supplementary Figure S10.tif]

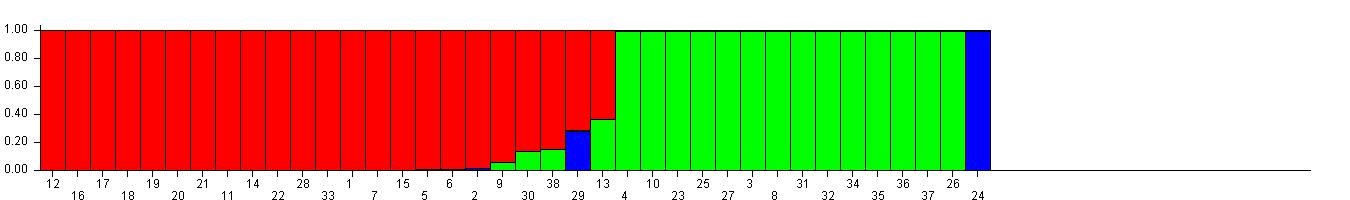

Supplement: Supplementary file 1 [file plants-13-00088-s001.zip › plants-2584093-supplementary/Supplementary_Files/Supplementary Figure S11.jpg]

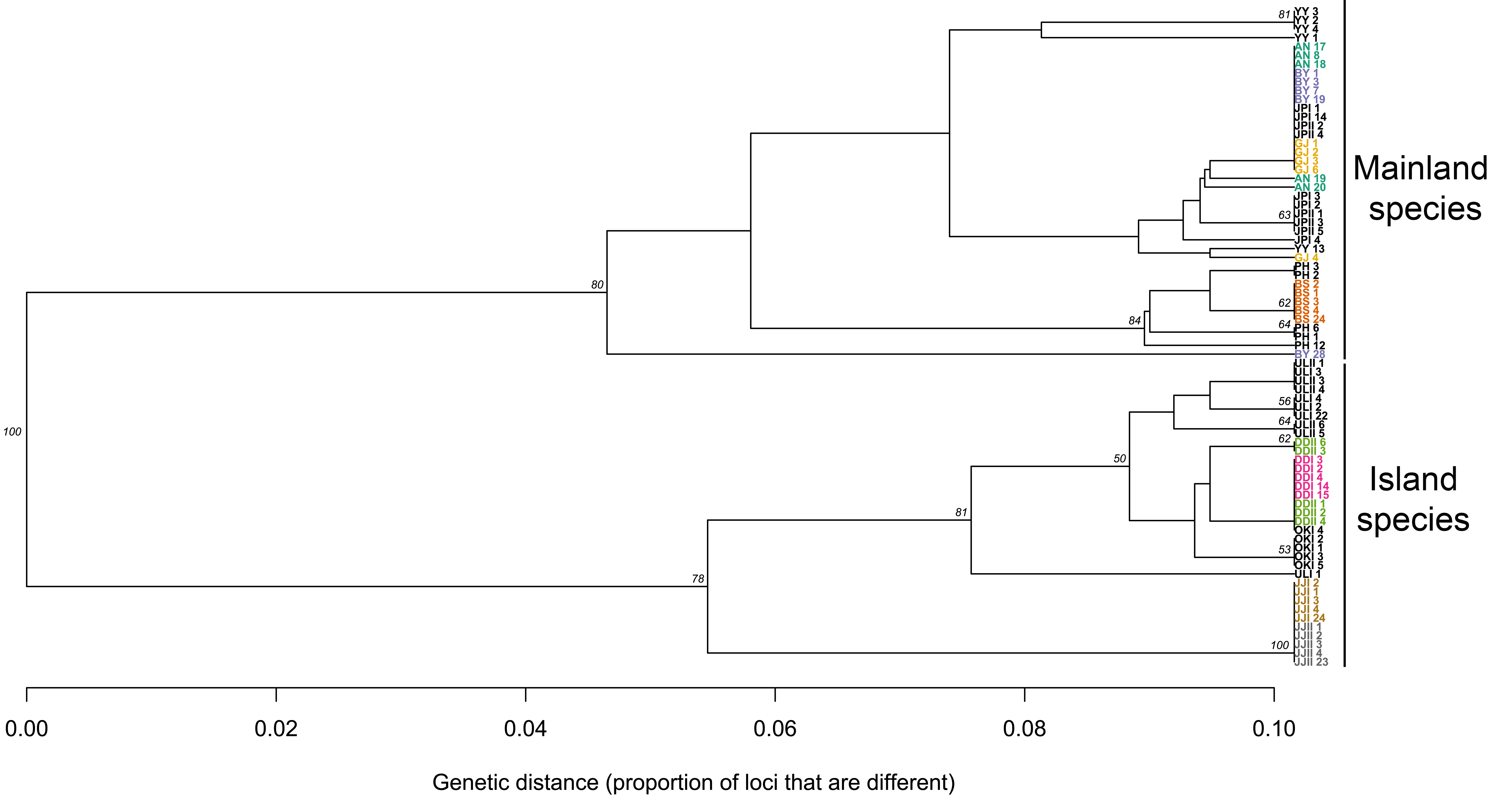

Supplement: Supplementary file 1 [file plants-13-00088-s001.zip › plants-2584093-supplementary/Supplementary_Files/Supplementary Figure S17.tif]

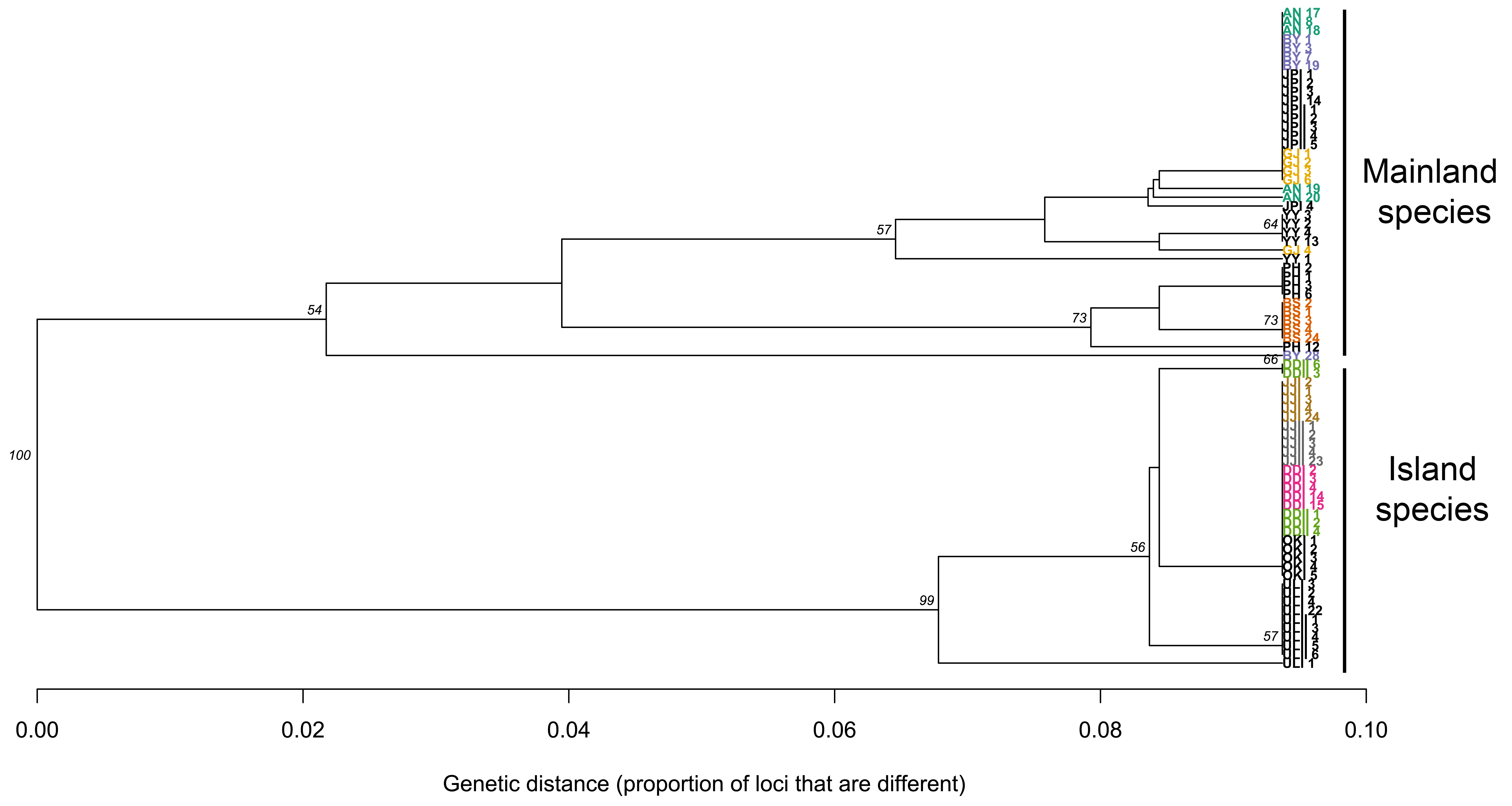

Supplement: Supplementary file 1 [file plants-13-00088-s001.zip › plants-2584093-supplementary/Supplementary_Files/Supplementary Figure S18.tif]

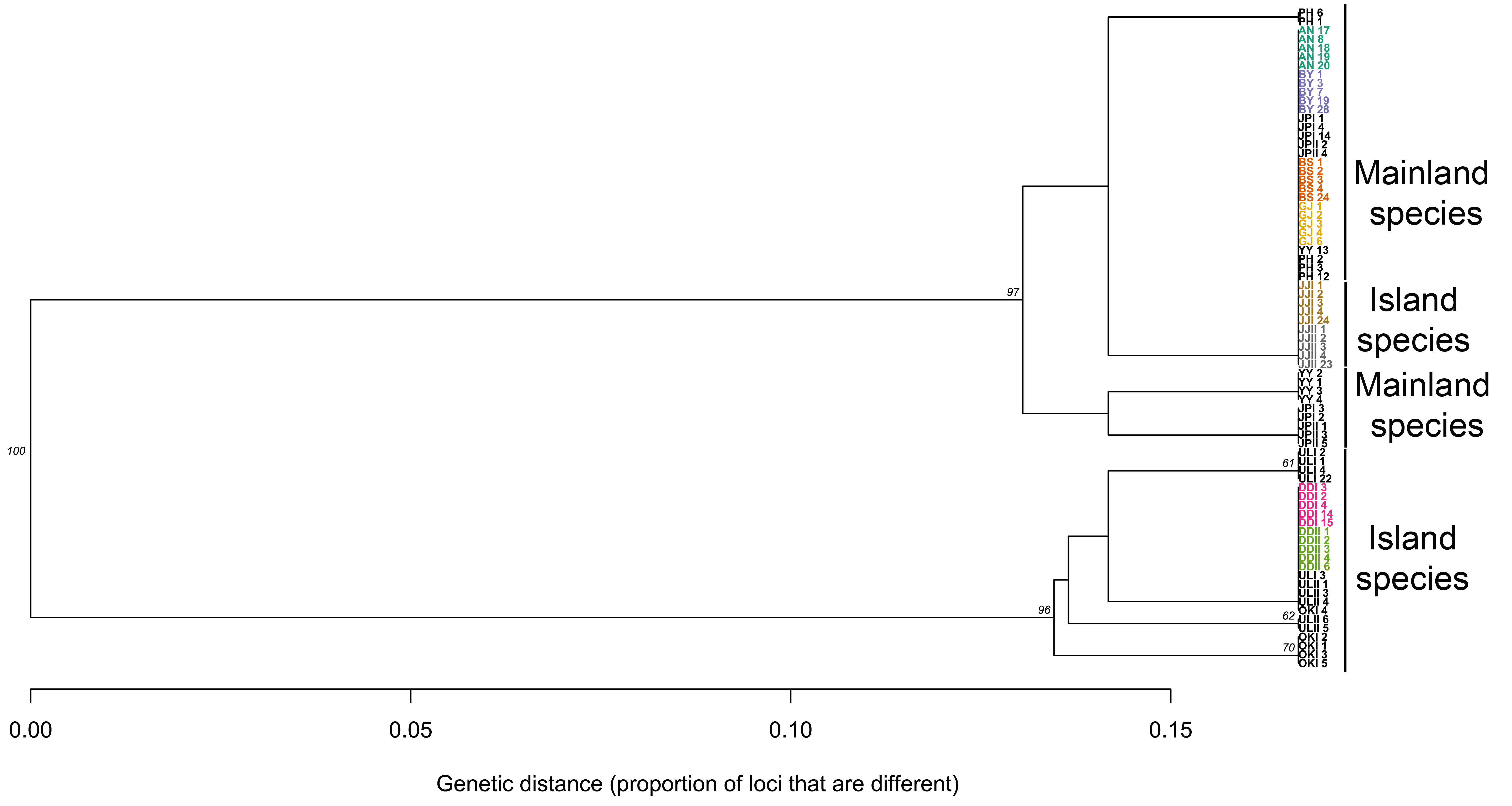

Supplement: Supplementary file 1 [file plants-13-00088-s001.zip › plants-2584093-supplementary/Supplementary_Files/Supplementary Figure S19.tif]

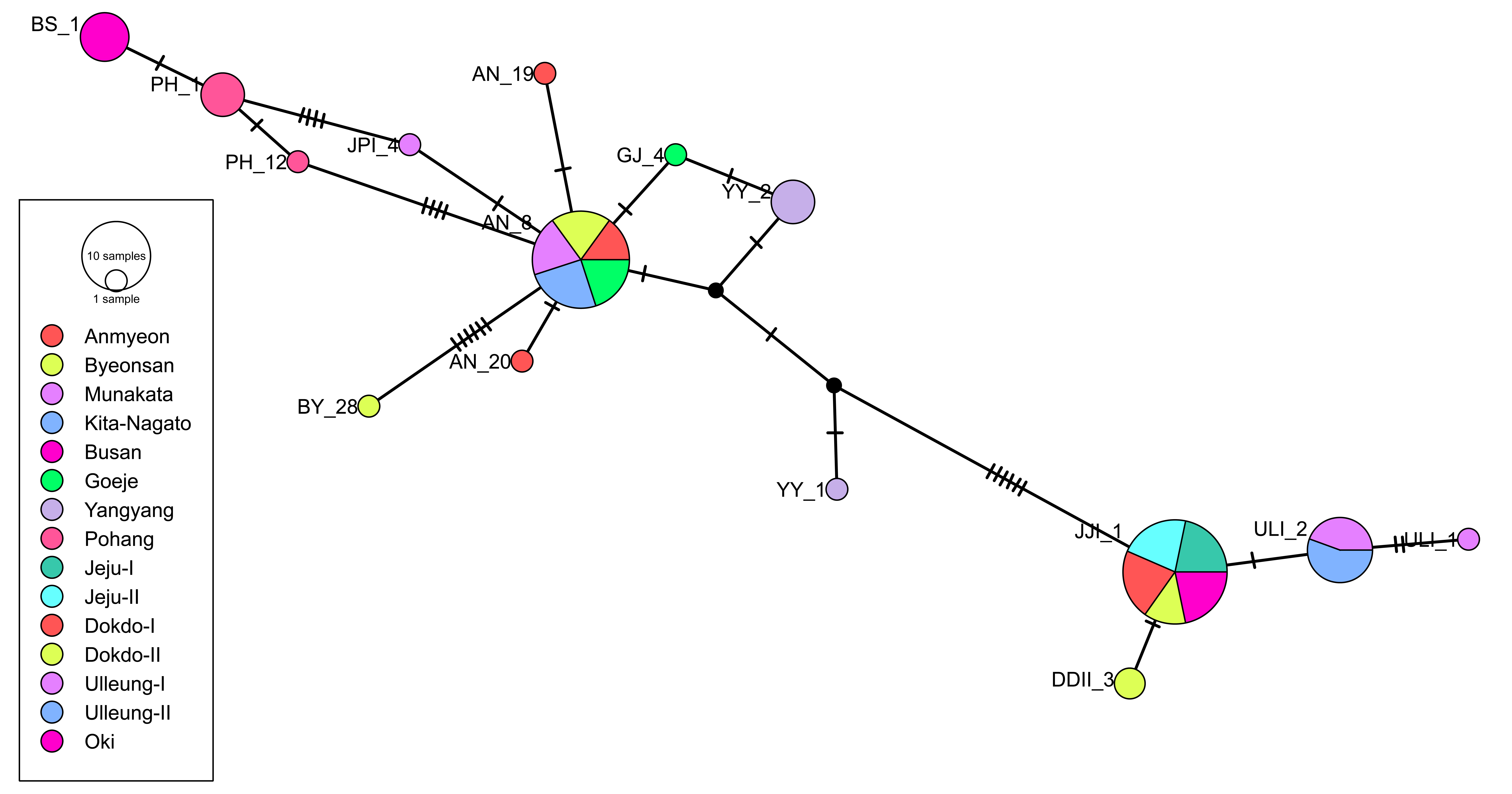

Supplement: Supplementary file 1 [file plants-13-00088-s001.zip › plants-2584093-supplementary/Supplementary_Files/Supplementary Figure S2.tif]

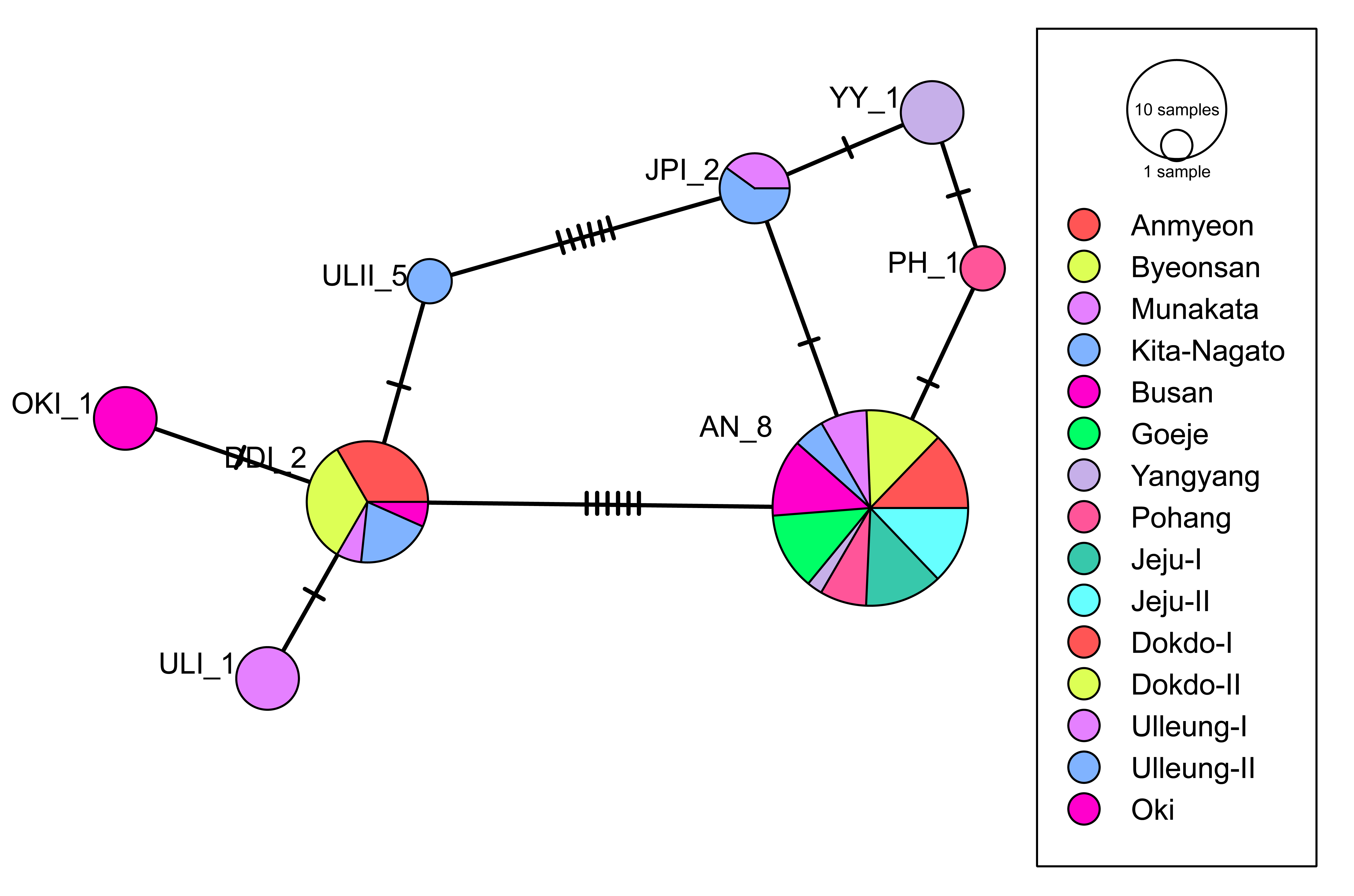

Supplement: Supplementary file 1 [file plants-13-00088-s001.zip › plants-2584093-supplementary/Supplementary_Files/Supplementary Figure S3.tif]

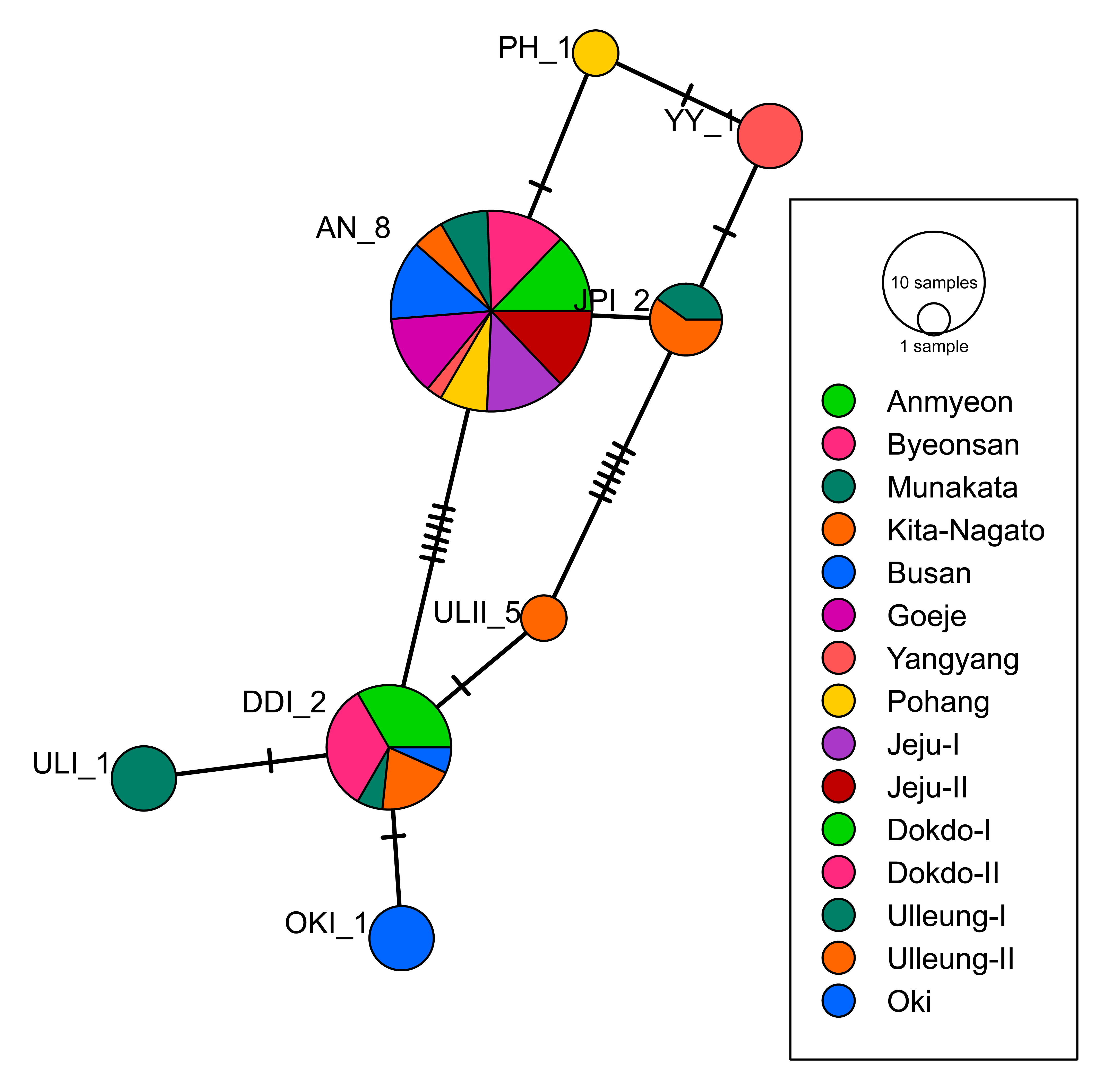

Supplement: Supplementary file 1 [file plants-13-00088-s001.zip › plants-2584093-supplementary/Supplementary_Files/Supplementary Figure S4.tif]

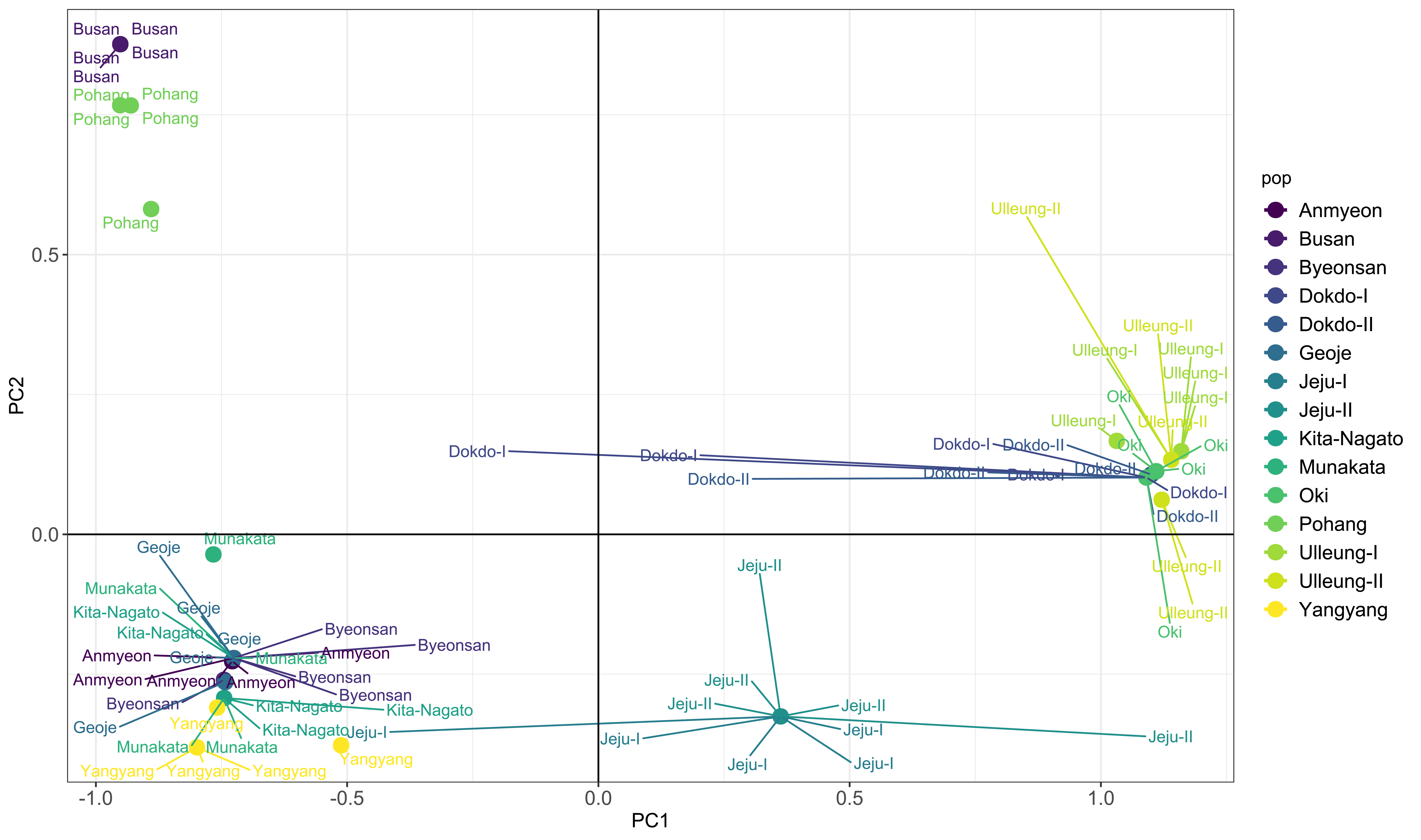

Supplement: Supplementary file 1 [file plants-13-00088-s001.zip › plants-2584093-supplementary/Supplementary_Files/Supplementary Figure S5.tif]

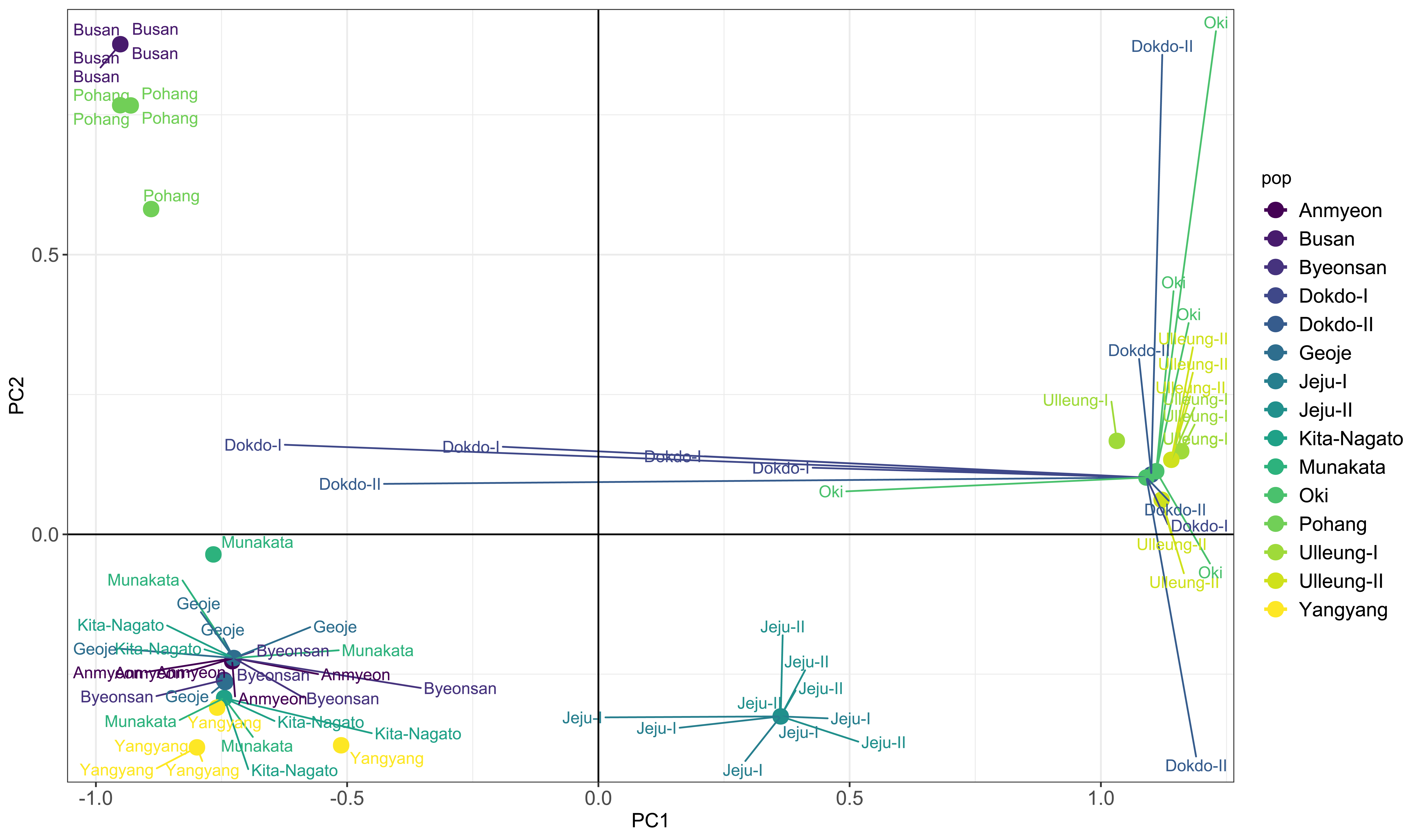

Supplement: Supplementary file 1 [file plants-13-00088-s001.zip › plants-2584093-supplementary/Supplementary_Files/Supplementary Figure S6.tif]

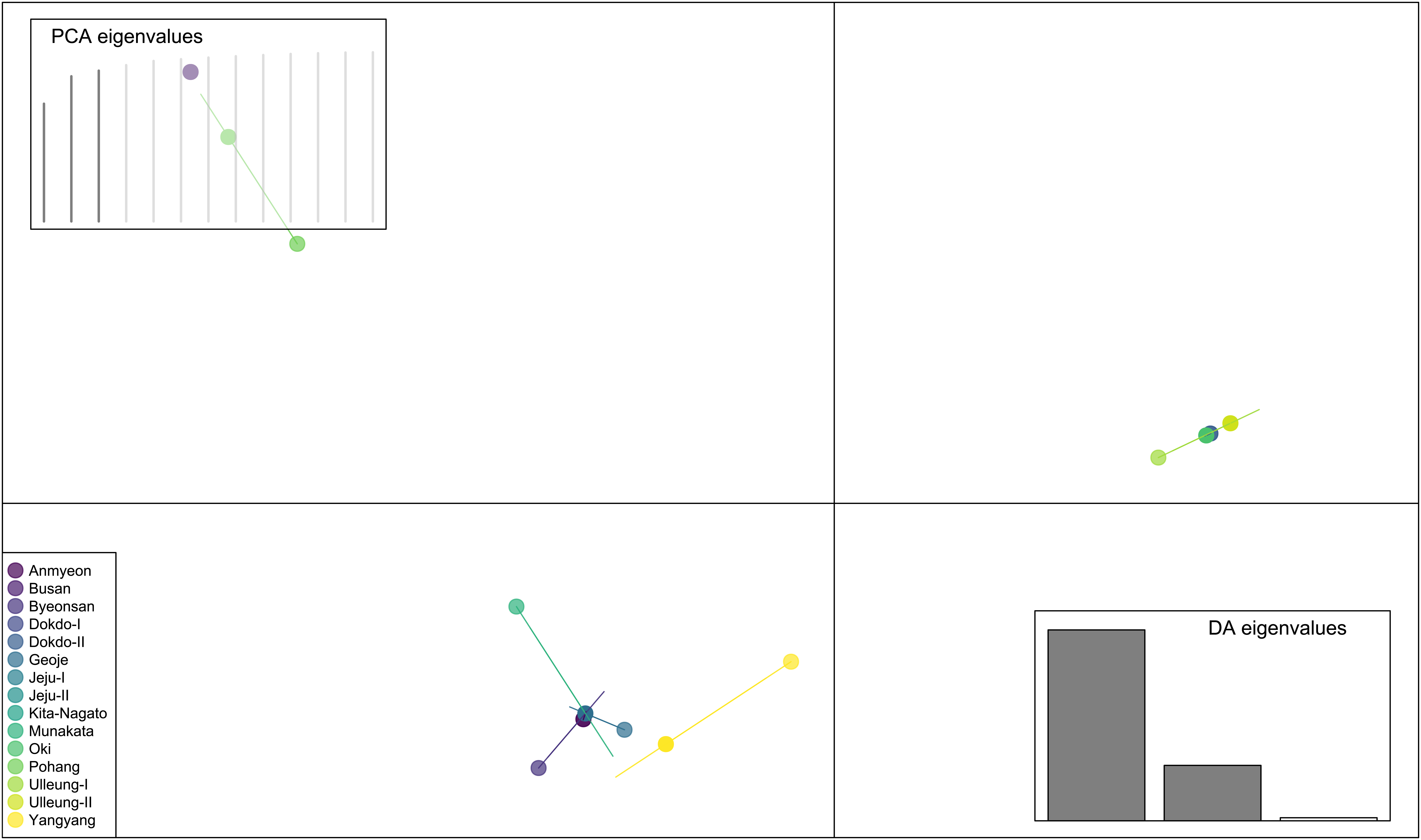

Supplement: Supplementary file 1 [file plants-13-00088-s001.zip › plants-2584093-supplementary/Supplementary_Files/Supplementary Figure S7.tif]

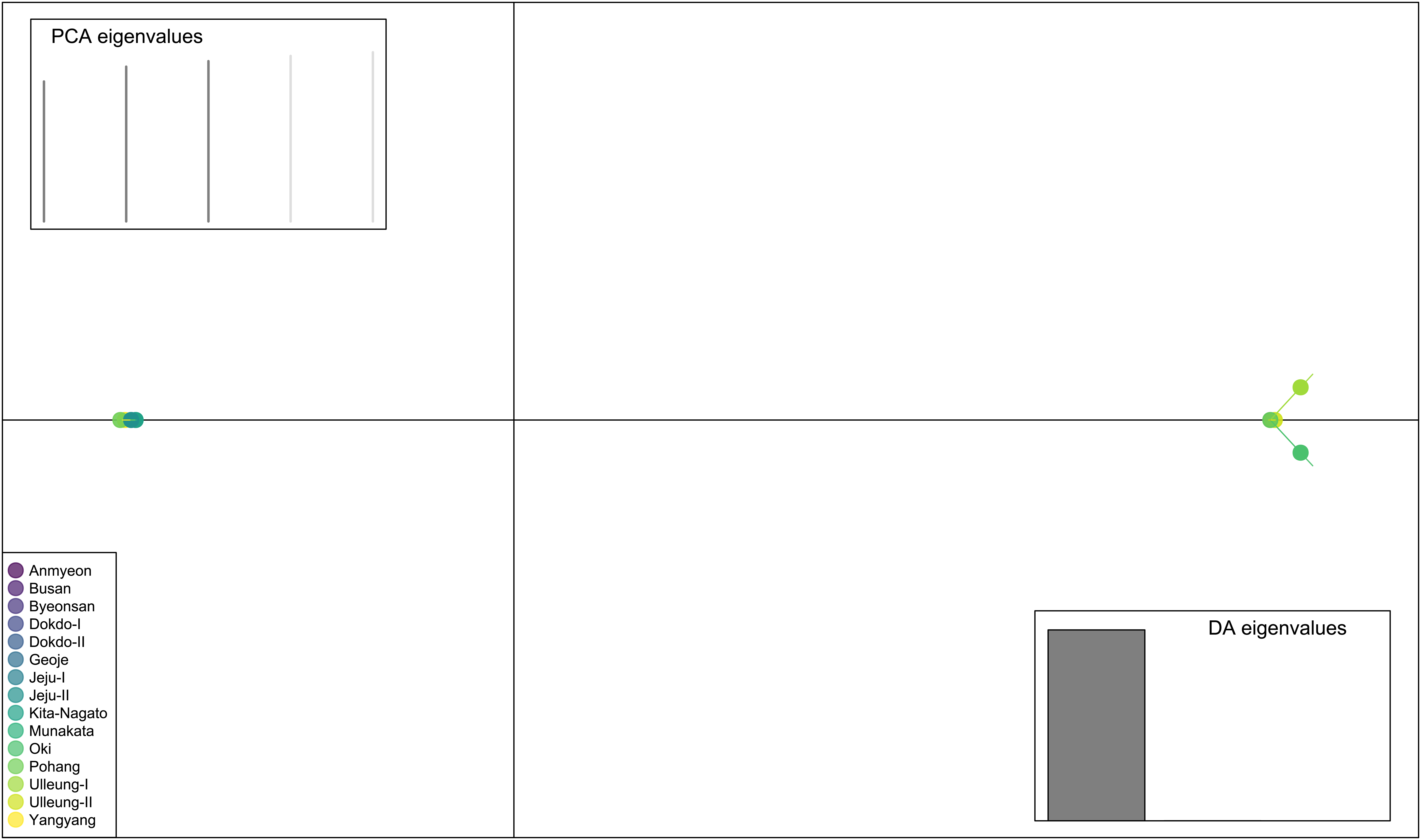

Supplement: Supplementary file 1 [file plants-13-00088-s001.zip › plants-2584093-supplementary/Supplementary_Files/Supplementary Figure S8.tif]

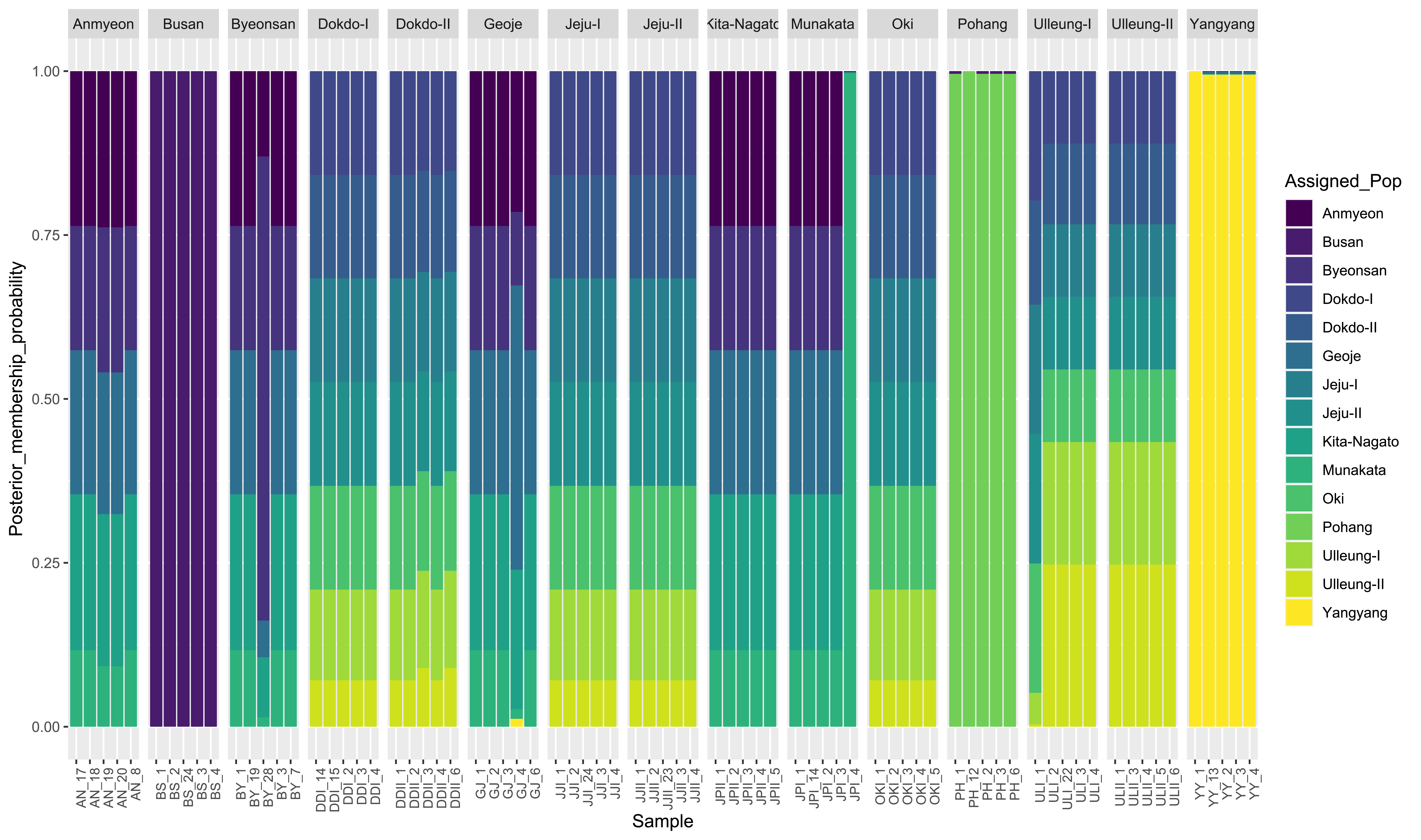

Supplement: Supplementary file 1 [file plants-13-00088-s001.zip › plants-2584093-supplementary/Supplementary_Files/Supplementary Figure S9.tif]
